# From dairy to plant products: Understanding their structural fingerprints with X-rays

**DOI:** 10.1038/s41538-025-00493-w

**Published:** 2025-06-23

**Authors:** Eleonora Olsmats, Adrian R. Rennie

**Affiliations:** Macromolecular Chemistry, Department of Chemistry – Ångström, Uppsala University, Box 538, 75121 Uppsala, Sweden

**Keywords:** Characterization and analytical techniques, Soft materials

## Abstract

Global interest in milk alternatives increases rapidly due to health awareness, their allergen-friendliness, and concerns about sustainability. While dairy product microstructure and rheology are widely studied, plant-based alternatives remain less understood, with limited comparative studies of different plant sources and brands. This study uses ultra-small, small and wide-angle X-ray scattering (USAXS, SAXS, WAXS) to analyse structural fingerprints of commercial plant-based milk, yoghurt and cream alternatives versus dairy products. These techniques allow characterization across multiple length scales from large oil droplets and aggregated structures to carbohydrate/protein networks and glyceride crystalline phases. Correlations between intensity and fat (USAXS) and carbohydrate content (SAXS) provide structural insights, while SAXS and WAXS data correlated with solid fat and crystal packing are important for melting behaviour and viscosity perception. Light scattering confirmed fat-content-related size trends and revealed larger structures of non-lipid materials in plant-based samples. The study provides a basis for understanding scattering data where structural fingerprint plots, using colour scales to compare intensity and intensity gradient, allow ready data interpretation that will be beneficial for analysis with artificial intelligence (AI) tools. This approach helps optimize plant-based formulations by connecting structure and functionality and demonstrates the potential of scattering techniques in food structure research and design.

## Introduction

The global demand for plant-based milk and yoghurt-like alternative products has grown significantly in recent years, driven by increasing consumer interest in sustainable, lactose-free and allergen-friendly options^1^. Products derived from almond, coconut, fava bean, linseed, oat, pea, rice and soy are now widely available, offering a range of nutritious and sensory choices.

One of the main challenges in developing plant-based dairy alternatives is to replicate the texture and mouthfeel of traditional dairy products. Mouthfeel is influenced by factors such as viscosity, particle size distribution, possible melting of crystalline structures, as well as emulsion stability. Physics plays an important role in understanding and optimising these properties. For example, dairy milk is a stable emulsion of fat globules and casein proteins, which contribute to the creamy texture. In contrast, plant-based alternatives often require additional structuring agents, such as polymers or emulsifiers to achieve similar textural properties.

Physical models, particularly in rheology, are used to relate factors such as shear thinning behaviour and yield stress to mouthfeel and texture. These models describe flow under different conditions and can be applied directly to understand how consumers perceive a texture. Examples of such models used in this context include the Herschel-Bulkley and Casson models that both describe yield stress materials^2^. The yield stress is the minimum stress required to initiate flow in a material. Many food products such as mayonnaise and chocolate exhibit yield stress behaviour as they behave like solids until a critical stress is applied, after which they start to flow like a liquid. Advanced approaches, such as computational fluid dynamics simulations allow for detailed analysis of flow and emulsion stability based on the preparation procedure^3^. Temperature also plays a critical role in the rheological behaviour as it affects both the viscosity and emulsion stability, which are important for the consumer sensory perception and overall acceptability. Understanding how these rheological properties are connected to the physical structure not only enables improvements in texture and mouthfeel but can also be used to make health-related choices related to, for example, fat content and digestibility.

Intake of traditional dairy milk and yoghurt products has been extensively studied by Michaëlsson and colleagues^4^. They found a greater risk of ischaemic heart disease and acute myocardial infarction for women with daily non-fermented milk intakes greater than 300 ml. It is possible that this effect is not present in plant-based alternatives due to the absence of lactose (galactose), as no effect was seen for fermented dairy milk products where the galactose has been degraded by fermenting bacteria. Another interesting observation is that these results were unrelated to fat content, which is thought-provoking from a view of plant-based alternatives, which often contain less fat due to calorie concerns. The conventional consumer assumption is that a plant-based product can be used directly as a replacement for dairy products, however, a closer look at their nutritional values reveals that the energy balances are very different^5^. For nut-based products such as those derived from almond and coconut, a majority of the energy is from fats, whereas for cereal-based products such as those made of oat and rice materials, the carbohydrate content is dominant. Dairy products tend to have a more balanced distribution of macronutrients. To our knowledge, a human consumption health study of the scale of that by Michaëlsson et al.^4^ has not been conducted for plant-based alternatives, but other studies have indicated various health benefits of diets with plant-derived foods^6,7^.

The composition of plant-based alternatives is diverse. Products made from almond, coconut and soy rely primarily on the fat, carbohydrates and proteins naturally present in the crop, while products based on oat, pea and rice often incorporate additional fats or oils from other sources to obtain the desired compositions.

A further reason for the addition of extra materials to the plant-based alternatives are the questionable flavour profiles. Common approaches to address this issue are either to add sweeteners or artificial flavouring ingredients, or by using fermentation procedures^8^. The use of fermentation is seen in many of the yoghurt mimicking dairy products on the market, where microbes such as lactic acid bacteria have been added.

Dairy products, consisting of casein micelles and lipid globules, have been extensively studied with techniques such as rheology and scattering, and the structural properties have been correlated with properties such as stability and texture^9–14^. Hierarchal structures are commonly seen of micrometre sized fat globules, casein micelles of the order 1000 Å, colloidal calcium phosphate nanoparticles of a few tens of Ångström, and many micrometres sized fat crystals with internal structures giving rise to correlations of a few Ångström. Despite the popularity of the plant-based alternatives, the structural and functional properties remain less understood. A previous comparative study of dairy and plant-based products describes more inhomogeneous structures and large clusters of gelatinised starch and big protein particles in the plant-based materials^15^. However, detailed analysis is limited and there was no direct connection made between structural description and physical properties. Understanding the microstructure of these formulations better is essential for improvement of product composition and functionality. Challenges with these products are the complexity – they do not contain only lipid droplets and emulsifier but other materials such as dispersed proteins and carbohydrates that can interact in many ways to form various different structures. These highly polydisperse samples with many components, influenced by both ingredient selection and processing methods, present significant challenges for characterisation.

Small-angle scattering techniques, such as ultra-small-angle X-ray scattering (USAXS), small-angle X-ray scattering (SAXS) and wide-angle X-ray scattering (WAXS) are powerful techniques to investigate hierarchical structural properties in a non-destructive manner. These complementary techniques allow measurements over length scales from micrometre lipid droplets and aggregated materials (USAXS), protein particle size and fractal structures (SAXS), down to the packing of individual molecules at Ångström scales (WAXS). Scattering arises from all inhomogeneities in a sample providing information about mixing of components and structure. These methods were complemented by dynamic light scattering (DLS) that can provide a better indication of the largest dimensions such as the overall size of emulsion droplets. Together, these techniques provide a comprehensive view of structural organisation from molecular arrangement to micron droplet sizes. Ultimately, the structure can be related to physical properties.

In this study, we performed USAXS, SAXS and WAXS measurements to analyse the structural properties of a range of commercial plant-based milk and yoghurt alternatives, to compare with dairy products. Our aim was to explore the diversity of these systems in terms of structural features and to show the potential of these technique for comparative analysis with many samples even without direct modelling. This study is unique and useful in the sense that by comparing a range of different plants as well as products from different brands, we can present a direct comparison of these products and put them into context. Scattering work on these types of plant-based emulsions are surprisingly rare and provides extra novelty to this study. We present these data as fingerprint maps for each sample on a colour heat scale, as an easy but sophisticated tool to visualise and derive preliminary interpretation of scattering results. These structural fingerprint plots allow for easy visualisation of both the scattering intensity and the gradient for a large number of samples simultaneously and can cover the wide range of length scales investigated. The idea is that this would strongly benefit the food science community in the aim for better material characterisation. While the complexity of these systems makes quantitative modelling challenging, the scattering data reveals important qualitative trends and allows for identification of correlations that provide insights into the microscale and nanoscale organisation of plant-based products. We further relate the small-angle scattering results to complementary measurements of particle size, zeta potential and pH. The implications of these findings are related to the stability, texture and visual appearance, which are important parameters for a good product formulation. With this study we explore the structure of these various products, however, the study should also be viewed as an example of how scattering techniques can be applied to future studies in related fields including foods, pharmaceuticals and other complex materials for structure characterization where direct modelling is challenging.

## Results

### Size distribution & visual appearance

**Fig. 1:**
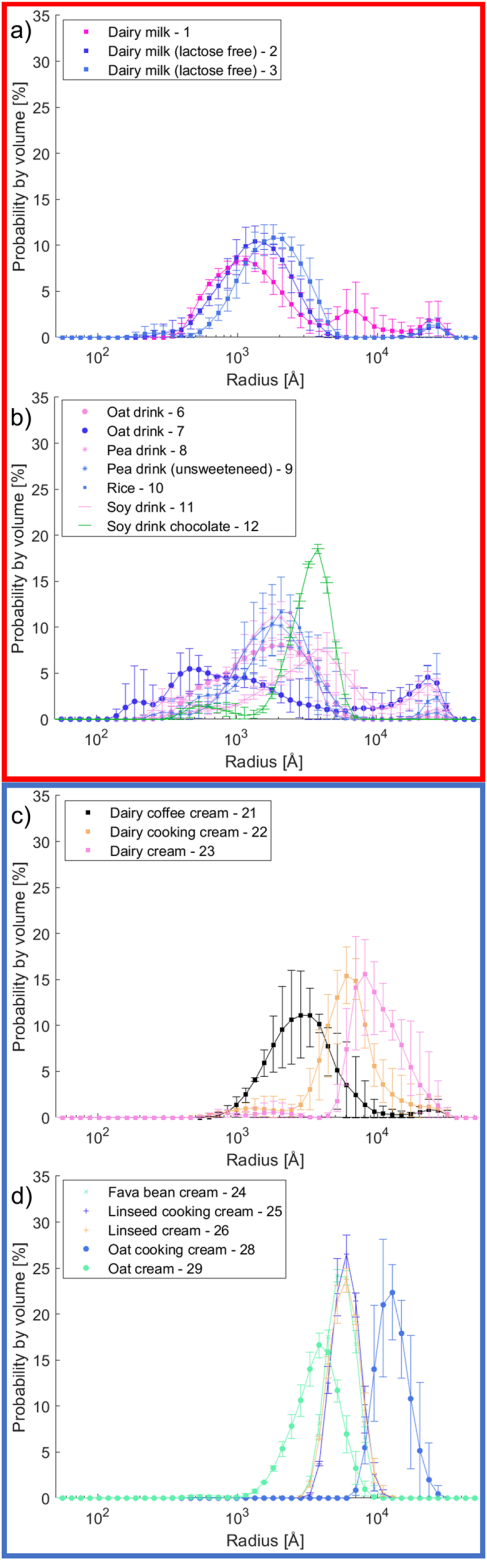
Distributions of radii for selected products obtained from dynamic light scattering. **a** Conventional dairy milk, **b** plant-based milk, **c** conventional dairy cream, and **d** plant-based cream products. Error bars show variation between measurements performed in triplicate.

The size distributions obtained by DLS are shown in Fig. 1 with corresponding plots of the correlation functions in Fig. S1 and median radii in Table S1 in the Supplementary Material. Although DLS typically reports sizes as diameters, in this study, the values are presented as radii in Ångström to facilitate comparison with X-ray scattering data discussed in the following paragraphs. DLS is well-suited to measure particle sizes in the nanometre range, however the accuracy is not good for the micron range due to longer correlation times required for slow-diffusing large objects and possible sedimentation. Nevertheless, DLS provides an idea about the overall size distributions and approximate dimensions of the objects. Some complications can arise with multicomponent systems where apparent distribution of size may reflect either the presence of different components or the same material dispersed or aggregated in different ways. A further issue arises with the use of the Stokes-Einstein equation to calculate the hydrodynamic size. Many of the plant-based materials are not necessarily spheres and rotational diffusion or other motion can contribute to the decay in time correlation and hence lead to inaccurate size estimates from single angle measurements. Additionally, it is possible that some of the materials in the plant-based samples are dissolved in the continuous phase, contributing to altered viscosity and refractive index of the medium. Interpretation of data depends on the assumptions made for the refractive index of the components. A refractive index for the dispersed material of 1.45^16,17^ was assumed for the analysis of all data presented. Only results for the samples that showed reasonable time correlation functions are presented, excluding the yoghurt samples with high turbidity.

Comparing the distributions of conventional dairy milk (Fig. 1a) and plant-based milk supplements (Fig. 1b), clear differences are observed. While we see similar log-normal distributions of radius for the animal milks with median ~1500 Å, milk-like products based on cereals, legumes and nuts show objects with multimodal size distributions. The oat and soy drinks show a broad distribution of small particles of hundreds of Ångström to bigger clusters of tens of thousands of Ångström, with the exception being the chocolate flavoured soy drink. The chocolate flavoured drink has a significantly higher sugar content compared to the natural soy drink, which could form complexes with the proteins and hence create a more defined size distribution. The drinks based on pea and rice replicate very well the dairy milk samples with broad log-normal distributions of radii with median around ~2000 Å.

The visual appearance, or brightness, of a milk-type product is related to the scattering of light by the dispersed material. Generally, the scattering is highest from objects with radius ~1000 Å, and increases for higher concentrations to a certain level^18^. The white appearance arises due to scattering from regions with different refractive indices that are larger than the wavelength of visible light. For casein-based products (Fig. 1a), this would have contributions from both oil droplets and casein micelles, which give the characteristic opaque and white appearance. For plant-based alternatives, with less defined structures, it is not surprising that it is an issue to produce products that appear as white. For oat products in particular, it is possible that some natural pigments are contributing to the brownish colour. Photographs of the products used in this study are presented in Fig. S2 in the Supplementary Material. With this in mind, it is tempting to see the potential of pea and rice-based products with distributions of radii more closely corresponding to that of conventional milk. These are likely to be appealing for the consumer with an expectation of the white appearance of standard milk.

**Fig. 2:**
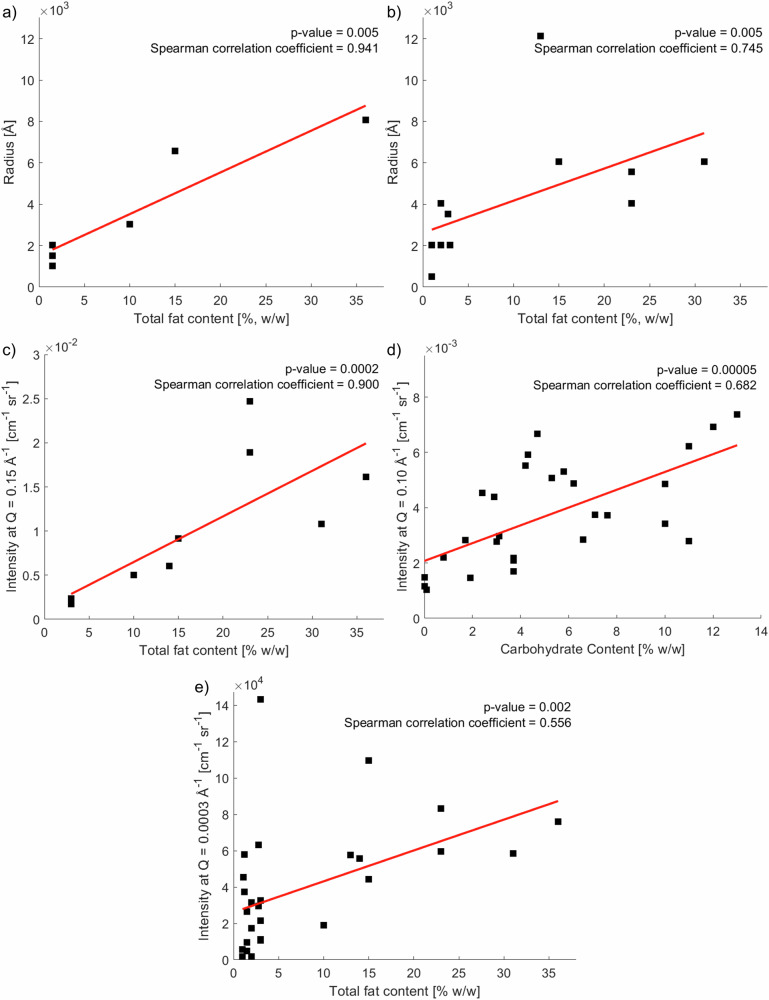
Correlations between sample composition and scattered intensity. **a** Median droplet radius of the volume weighted distributions from DLS versus total fat content for dairy samples. **b** Median droplet radius of the volume weighted distributions by DLS versus total fat content for plant-based samples. **c** SAXS intensity at *Q* = 0.15 Å^−1^ corresponding to the peak arising from fat crystallisation versus total fat content for relevant samples **d** SAXS intensity at *Q* = 0.10 Å^−1^ versus carbohydrate content. **e** USAXS intensity at *Q* = 0.0003 Å^−1^ versus total fat content. *p*-values for the Spearman correlations are <0.05. These suggest that the null hypothesis of no correlations can be rejected.

Comparing the products in the cream category, the casein-based samples (Fig. 1c) have a log-normal distribution of radii similar to milk, but the size is greater for higher fat concentrations. This is simply because more of the bigger oil droplets are contributing to the scattering signal. The contribution from the casein micelles is also seen as a smaller peak at about 1500 Å, but this is almost entirely hidden by the contribution from oil droplets. A plot of the centre of the distribution versus fat content for all dairy products is presented in Fig. 2a, where the radius increases linearly with fat content. A clear correlation is observed. However, this is not as apparent for the plant-based products in Fig. 1d. The smaller structures, in Fig. 1b, are not present for the samples in Fig. 1d. The fitting of a straight line to the distribution average versus fat content is not perfect (Fig. 2b), however a Spearman correlation coefficient of 0.75 and a *p*-value of 0.005 for a null hypothesis of no correlation still indicates that there is a significant correlation. The lack of a perfect fit suggests that the other material (carbohydrates and proteins) in the vegetable dairy substitutes are forming large structures on similar micrometre length scales as those of the oil droplets, contributing to the scattering from large objects.

While many of the plant-based products in the milk category (Fig. 1b) have bimodal or multimodal distributions of radii and hence the simple polydispersity index (PDI) may be misleading, the plant-based cream products show a single peak. The PDI of these are around 0.1. This is lower than the dairy cream products (Fig. 1c) of 0.2–0.5. To understand this, it is also interesting to investigate the zeta potential and how this may be important in the stabilisation mechanism and establishment of electrostatic interactions to avoid aggregation.

### Charge effects

**Fig. 3:**
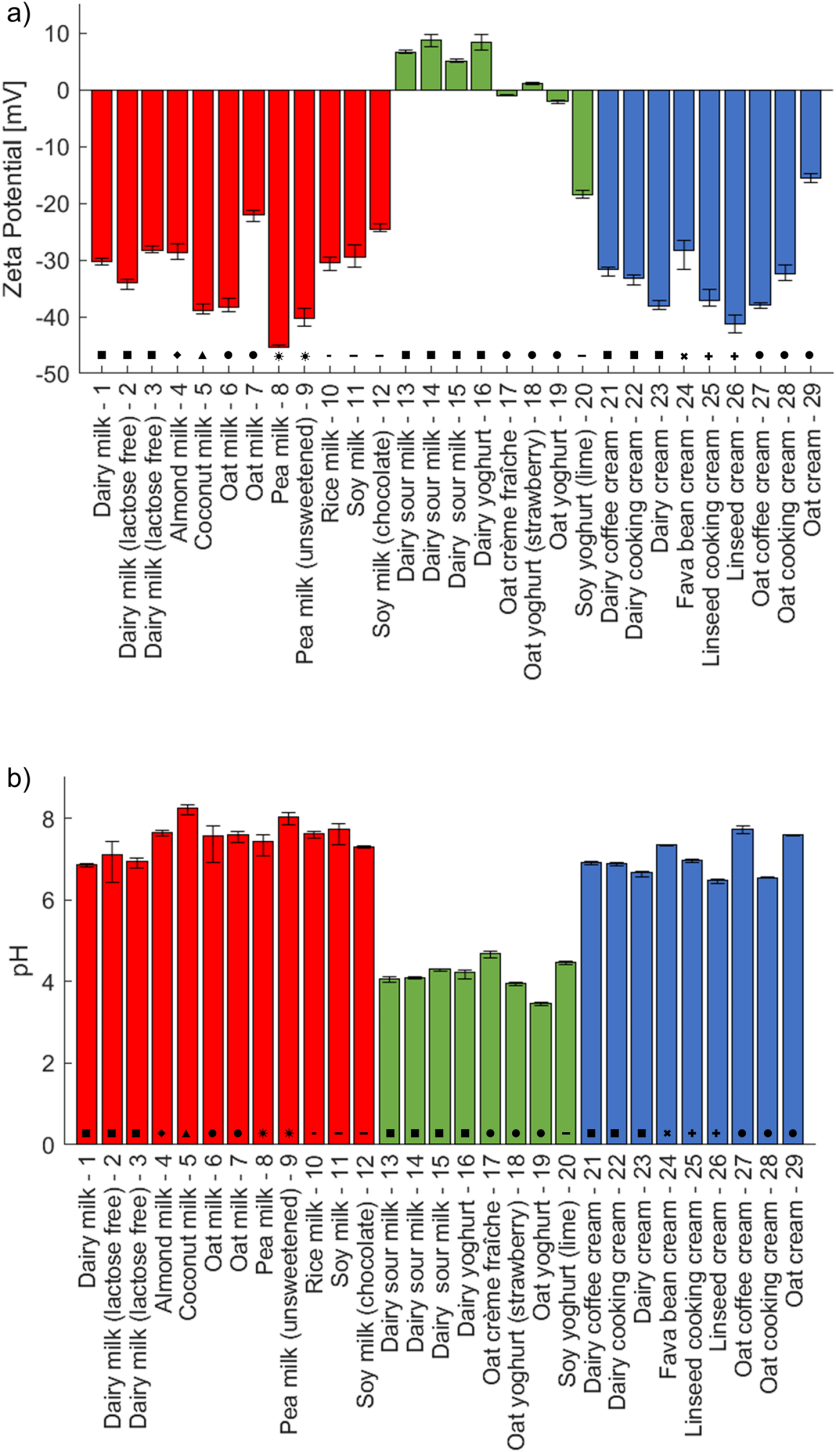
Zeta potential and pH for the samples. **a** Zeta potential and **b** pH. The bar colours represent milk (red), yoghurt (green) and cream (blue) products and the symbols correspond to the different materials described in the Materials section.

The zeta potential for all samples is plotted in Fig. 3a. This property is related to the electrophoretic mobility of the materials in a liquid where both the surface charge and the distribution of counter-ions in the electrical double layer surrounding the material determine this potential. In general, a high absolute zeta potential indicates good electrostatic stabilisation in colloidal systems. However, salt concentration is very important for stability, as higher salt concentrations (ionic strength) cause screening of the electrostatic interactions, compress the electrical double layer, reduce the zeta potential and promote aggregation^19^. The sodium chloride content in the samples varied between 0.03 and 0.25% w/w, corresponding to a molarity of 5–43 mM. Most of the samples also contained calcium (about 0.1% w/w) as carbonates, citrates or phosphates. Freely dissolved divalent calcium ions could contribute significantly to destabilization by screening electrostatic interactions and cause aggregation. The solubility of calcium carbonate at neutral pH is lower than 0.1% w/w^20^. However, it is possible that the pH of the fermented products (yoghurt category) is sufficiently low that the increased amount of soluble calcium significantly influences any electrostatic interactions by reducing the Debye screening length to a few Å. This is, for example, relevant particularly for the ‘Oat yoghurt - 19’ sample where calcium phosphate and calcium carbonate are added. The latter would have a solubility close to 5% w/w (molarity 0.46 M) at pH 4.5.

A high absolute zeta potential is observed particularly for the pea, coconut and linseed products, as well as in some cases of oat and dairy based materials. The green bars, representing yoghurt products, are samples with zeta potential around zero, suggesting negligible electrostatic contribution to the stability that would also be reduced by more dissolved calcium ions. A pH of 4 to 5 for these products is close to the isoelectric points of the proteins and lowers their solubility. The lower pH is due to the conversion of some sugars to acids when fermenting agents are added. The pH for all samples are shown in Fig. 3b. An interesting observation is the negative zeta potential of the ‘Soy yoghurt (lime) - 20’ sample despite the acidic pH. The likely explanation is the addition of pectin, which is negatively charged at this pH and interacts with soy proteins to increase the overall negative charge of the dispersed material. This is a similar effect to that shown with addition of pectin to casein aggregates in acidified dairy products that lowers the zeta potential due to adsorption driven by electrostatic interactions^21,22^. The negatively charged pectin molecules with ionized carboxyl groups adsorb to the surface of the positively charged casein aggregates with protonated amine groups and neutralise the charge in such products.

There is no general difference in zeta potential for the dairy and plant-based cream products. The ‘Oat cream - 29’ showed the lowest net zeta potential among these materials. This sample contains added polyethylene glycol sorbitan monostearate (Tween 60) that is known to decrease the net zeta potential as the surfactant concentration increases due to changes in the interfacial layer formed^23^. This surfactant is also present in the ‘Fava bean cream - 24’, a possible explanation for the zeta potential above −30 mV despite the high pH.

### X-ray scattering

Scattering curves from USAXS, SAXS and WAXS experiments cover a range of momentum transfer, *Q*, of 0.0003–3 Å^−1^, corresponding to structures from about 2 Å to 2 µm. With this broad range of length scales, it is possible to detect the hierarchy of structures from atomic levels to those of proteins, starch networks and fat globules.

### WAXS

**Fig. 4:**
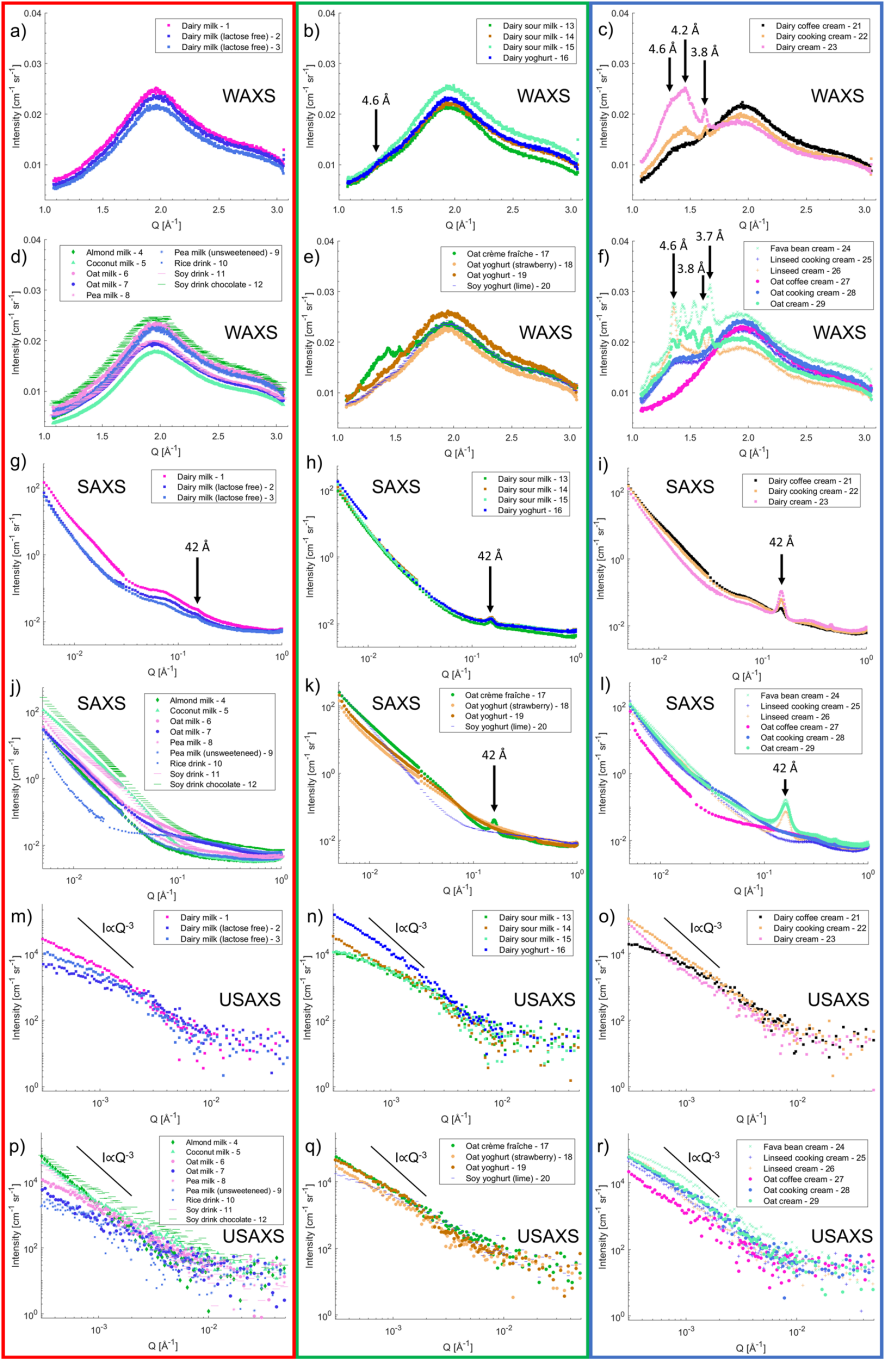
WAXS, SAXS and USAXS data for dairy and plant-based products. WAXS data: **a** Dairy milk products, **b** dairy yoghurt products, **c** dairy cream products, **d** plant-based milk products, **e** plant-based yoghurt products, **f** plant-based cream products. Arrows mark the *d*-spacing corresponding to the Bragg peaks observed in the scattering. SAXS data: **g** Dairy milk products, **h** dairy yoghurt products, **i** dairy cream products, **j** plant-based milk products, **k** plant-based yoghurt products, **l** plant-based cream products. The arrow at *Q* = 0.15 Å^−1^ mark the first order lamellae peak, suggesting a repeat distance of 42 Å. USAXS data: **m** Dairy milk products, **n** dairy yoghurt products, **o** dairy cream products, **p** plant-based milk products, **q** plant-based yoghurt products, **r** plant-based cream products. The black solid lines have gradients of *Q*^−3^, corresponding to the Porod law behaviour for Bonse-Hart smeared USAXS data. A plot of intensity at lowest *Q* versus total fat content for all samples is shown in Fig. 2e.

Starting the analysis with the smallest structures (highest *Q* values), plots of the scattering results are presented in Fig. 4a–f, with individual plots of each sample for additional clarity provided in Figs. S3–S5 in the Supplementary Material. In this WAXS region, the scattering for all samples is mainly contributing to a broad peak typical of an amorphous structure, which is centred around 2 Å^−1^. This arises from the oxygen-oxygen bond distance in the water molecule of about 3 Å^24^. However, for some yoghurt and cream type samples, additional crystal peaks were observed, which are indicated by arrows in Fig. 4. There are three crystalline forms for simple triglycerides with three identical fatty acids – α (hexagonal), β′ (orthorhombic), and β (triclinic) crystals^25^. These configurations of the packing of hydrocarbon chains of the triglycerides can be observed as Bragg peaks in the WAXS region, where the positions are listed in Table 1^25–27^. Further details of peak widths, intensities and possible peak overlaps can be identified from the data files recorded in this study that are available in the repository described in the Data availability section.

**Table 1:** Bragg peak positions in the WAXS spectrum and corresponding *d*-spacing for the α (hexagonal), β′ (orthorhombic), and β (triclinic) crystals in triglycerides as reported^25–27^

Crystalline structure	Bragg peak position in *Q* [Å^−1^]	*d*-spacing in real space [Å]
α (hexagonal)	1.53	4.15
β′ (orthorhombic)	1.50 & 1.65, or 1.47, 1.58 & 1.69	4.2 & 3.8, or 4.27, 3.97 & 3.71
β (triclinic)	1.37, 1.65 & 1.70	4.6, 3.8 & 3.7

In general, conventional dairy milk contains no fat crystals, which we also confirm by the lack of diffraction peaks in the WAXS spectrum, but studies have shown that β (triclinic) crystals can form when the milk fat is entrapped in a protein gel matrix^28^. The fat globules are occupying the space between proteins and prevent protein aggregation. In a solid food matrix, such as that in cheese samples, the authors hypothesise that the triglycerides are forced to arrange in the most compact and stable form, favouring the formation of β (triclinic) crystals. We observed the formation of β (triclinic) crystals in the samples of ‘Dairy Yoghurt - 16’, ‘Dairy coffee cream - 21’, ‘Dairy cooking cream - 22’, ‘Dairy cream - 23’, ‘Fava bean cream - 24’, ‘Linseed cooking cream - 25’, ‘Linseed cream - 26’, ‘Oat cooking cream - 28’, and ‘Oat cream - 29’. The peak with *d*-spacing 4.6 Å is most clearly visible, and for most of these products we observe a broad peak, attributed to the many different types of configurations for these natural systems. However, for the ‘Fava bean cream - 24’, ‘Linseed cream - 26’ and ‘Oat cream - 29’, the peak has a smaller width and additional peaks corresponding to spacings of 3.8 and 3.7 Å are present, further demonstrating the presence of β (triclinic) crystals. For these products, the heat treatment has presumably converted the crystal structure to the stable β (triclinic) configuration or a favoured composition of saturated triglycerides has been chosen to obtain the desired solid structure. Generally, transitions occur from the α (hexagonal) to β′ (orthorhombic) and β (triclinic) with different melting points^29^. The specific combination of triglycerides determines the polymorphic tendency to the β′ (orthorhombic) or β (triclinic) configuration^30^. From a consumer perspective, the β′ (orthorhombic) form is generally preferred in margarines and spreads due to the smooth texture and good sensory quality created, whereas the coarser texture of β (triclinic) crystals are preferred in, for example, the chocolate industry^31^. We see β′ (orthorhombic) packing for the ‘Dairy cooking cream - 22’ and ‘Dairy cream - 23’ samples. This has been observed previously for milk fat, and the reason suggested is the large distribution of triglyceride species and a significant amount of palmitic acid^32^. Mattice and Marangoni have shown that hydrogenated shortenings are stable in the β′ (orthorhombic) configuration, whereas nonhydrogenated samples convert to β (triclinic) over time^33,34^. However, the study by Neves et al.^32^ saw a transition to the β (triclinic) state for a hydrogenated soybean oil after 90 days of storage. The products containing hydrogenated fats in this study include the ‘Oat crème fraîche - 17’, ‘Fava bean cream - 24’ and ‘Oat cream - 29’. All of these samples show additional peaks, not frequently associated with any of these configurations. The likely explanation is the heterogeneous character of these systems, with different fatty acid chains in the triglycerides giving rise to broad diffraction peaks. α (hexagonal), the least stable and less dense crystal packing, is not clearly identified in any of the samples.

It is not surprising that the fat crystallisation depends on the food matrix and the type of emulsifier. This has also been reported previously when comparing fat blends with the addition of sucrose esters and sorbitan fatty acid esters^35^ and mono and diglycerides and polyethylene glycol sorbitan monooleate (Tween 80)^36^. A further recent example is the study of the addition of stearic-palmitic sucrose esters to anhydrous milk fat where X-ray scattering and microscopy were used to study the fat crystallisation behaviour^37^. All the plant-based whipping cream alternatives in this study contain mono and diglycerides as emulsifiers, which are likely to control the crystallisation of the fat to obtain a more solid and foamable product. It is of interest to correlate the preparation method and thermal treatment with different crystal structures that would influence texture and other physical properties but such information is not available for the products in the present study.

Although most products contain calcium through the addition of phosphates, carbonates or citrates, the concentration of 0.11–0.12% w/w is apparently insufficient to give rise to diffraction peaks. This suggests that at least some fraction is present as dissolved material in the aqueous phase as discussed in the Charge effects section. The primary reasons for the addition of calcium are presumably to enhance nutritional value and to provide a whiter colour.

### SAXS

SAXS data corresponds to structures on length scales from 5 to 1000 Å. In general, the SAXS data exhibit only one or two distinguishable diffraction peaks per sample, and these peaks are relatively broad in *Q* as compared to the sharper and more numerous peaks in the WAXS data due to the lower degree of long-range structural order and variability in domain size. The SAXS data are presented in Fig. 4g–l with individual plots of each data set in Fig. S6–S8 in the Supplementary Material. The scattering peak at *Q* = 0.15 Å^−1^, marked with an arrow in the main figure, is a first order peak from a lamellar structure, arising from the thickness of the triglyceride lamellae. This suggests a repeat distance of 42 Å in a 2 L configuration^28^. The 2 L configuration is a bilayer structure and the distance corresponds to the stacks of the lamellae with twice the molecular chain length as the repeat in the longitudinal direction^38^. This feature is observed for the following products: ‘Dairy milk - 1’, ‘Dairy milk (lactose free) - 2’, ‘Dairy milk (lactose free) - 3’, ‘Dairy sour milk - 13’, ‘Dairy sour milk - 14’, ‘Dairy sour milk - 15’, ‘Dairy yoghurt - 16’, ‘Oat crème fraîche - 17’, ‘Dairy coffee cream - 21’, ‘Dairy cooking cream - 22’, ‘Dairy cream - 23’, ‘Fava bean cream - 24’, ‘Linseed cream - 26’, and ‘Oat cream - 29’. Fig. 2c presents a correlation plot of scattered intensity versus total fat content for these samples. The plot demonstrates that there is a high content of solid fats in these samples. The Spearman correlation coefficient was 0.90 and the *p*-value was 0.0002, suggesting that the null hypothesis of no correlation could be rejected. Several of the plant-based products listed contain hydrogenated fats, presumably to increase solidity and viscosity through crystallisation. The result is a smoother and “melt-in-the-mouth” experience. Shen et al. have investigated the melting behaviour of hydrogenated coconut and cottonseed oils compared to anhydrous milk fats^39^. The solid fat content was strongly affected by the fatty acid composition, particularly by the saturation and double bonds of the C_18_ fatty acids. Formulations are usually chosen to show some solidity at room temperature and melting at higher temperatures. The scattered intensity of this peak gives an indication of the crystal structure. First, the width of the peak, referred to as the peak broadening, is related to both the size of the crystals, their structural heterogeneity and their internal strain. In this case, we observe broader peaks for the plant-based products compared to the dairy samples. This increased breadth is not surprising as it may be attributed not only to smaller crystal domains and increased disorder, but also to the presence of a greater variety in the chain lengths of the fatty acids when different materials in the plant-based samples interact and create various different structures. Additionally, it is helpful to note that not all SAXS peaks are expected to occur precisely at 42 Å, as the variety of fats in the products have a distribution of lamellar spacings. Secondly, the peak intensity corresponds to the amount of crystalline material, where the high intensities for the ‘Fava bean cream - 24’ and the ‘Oat cream - 29’ are likely to be an effect of the high content of hydrogenated fats. The resulting properties of improved texture and firmness are likely a requirement to keep the good solid and foaming properties with the relatively low fat content of 23% w/w. This fat content is significantly lower than the other whipping cream products (‘Dairy cream - 23’ with 36% w/w fat and ‘Linseed cream - 26’ with 31% w/w fat) investigated in this study, meaning that a larger amount of solid fat derived from hydrogenated material is required. This can be seen as the two points significantly above the linear fit in Fig. 2c.

Other observations related to the SAXS results include the presence of broad peaks for most of the samples. In general, such features at different *Q* values correspond to structures or correlations with lengths given by $2{\rm{\pi }}/Q$. The widths of the peaks are related to the spread of the correlated distance between objects, the disorder, and the presence of different materials. The many different components make analysis challenging, however some estimates of size of the structures can be made. Particularly the oat and soy materials show a broad feature between 0.01 and 0.1 Å^−1^, attributed to the high contents of carbohydrates and proteins in these samples respectively.

The upturn in intensity upon looking from high to low in *Q*-space, is slightly different for different samples. For example, the ‘Almond milk - 4’, ‘Coconut milk - 5’, ‘Pea milk - 8’, ‘Pea milk (unsweetened) - 9’ and ‘Soy milk - 11’ show a low intensity down to *Q* values below 0.1 Å^−1^ before an upturn in intensity due to the low carbohydrate content. Carbohydrate-rich samples such as the ‘Rice milk - 10’, ‘Oat yoghurt strawberry - 18’, ‘Oat yoghurt - 19’ and ‘Oat cream - 29’ show an upturn already at higher *Q* values. The correlation between carbohydrate content and the intensity at 0.1 Å^−1^ is plotted in Fig. 2d, with a Spearman correlation coefficient of 0.68 and a *p*-value of 0.00005. The upturn in the scattering from oat products starting at higher *Q* values compared to the almond, coconut, pea and soy materials is presumably due to smaller structures of degraded carbohydrates resulting from sample preparation. A table of the power law gradients in different regions of *Q* is provided in Table S2 in the Supplementary Material.

### USAXS

A look at small momentum transfer, *Q*, reveals information about droplet size, total fat content and properties of the oil-water interface, and also identifies any bigger structures of proteins and carbohydrates. In USAXS experiments, the slit smearing in one direction changes the power of *Q* by 1, provided that the slit length is long compared to the measured value of *Q*. The mismatch in gradients between the raw SAXS and raw USAXS data arises from this phenomenon. USAXS data cover length scales of the order 0.1 to 2 µm. The slit smeared scattering patterns for the samples are presented in Fig. 4m–r with plots of each data set separately in Figs. S9–S11 in the Supplementary Material. Identifiable for all samples are a high intensity with no or little sign of turning over at the lowest *Q*. Some straightforward analysis can be made by observing the shape of the scattering curves. Gradients between −3 and −1 in a plot of logarithm of *I* versus logarithm of *Q* indicate scattering from fractal systems, which are characterised by self-similarity over certain length scales^40^. This is what is observed in most of the SAXS region. For a range of momentum transfer that is sufficiently large compared to the reciprocal of the dimensions of the scattering objects, a slope of −4 in a similar plot, indicates that there is scattering from a sharp interface. In emulsion systems, this Porod law is useful as it allows straightforward determination of the specific surface area, *S*:

1 $$S=\frac{A}{V}\cong \frac{I{Q}^{4}}{2{\rm{\pi }}{\left(\triangle {\rm{\rho }}\right)}^{2}}$$

where $\triangle {\rm{\rho }}$ is the difference in scattering length density (SLD) between the dispersed material and the surrounding medium. $A=4{\rm{\pi }}{r}^{2}$ is the area of one piece of dispersed material, and $V$ is the sum of the volumes of the different components per unit of dispersed material^41^. The black lines in Fig. 4m–r have a gradient of $I={Q}^{-3}$, indicative of a Porod law for slit smeared data. Some materials clearly show this behaviour, which is demonstrated in a plot of *Q* vs. *IQ*^*4*^ for desmeared USAXS data in Fig. S12 in the Supplementary Material. The specific surface areas and radii of these products were determined as 3600 cm^−1^ and 8.3 µm for ‘Almond milk - 4’, 1900 cm^−1^ and 15.7 µm for ‘Coconut milk - 5’, 4300 cm^−1^ and 6.9 µm for ‘Soy milk - 11’ and 5900 cm^−1^ and 5.1 µm for ‘Dairy cream - 23’. These calculations assume that the interfacial contrast arises between spherical droplets of oil and water, and hence an SLD of the oil of 8.7 × 10^−6 ^Å^−2^ was used for these calculations^42^. Although we do not observe a Guinier region to identify the dimensions for these big objects, the values give an indication of the overall size. The Guinier approximation in scattering data is a logarithmic variation of intensity with *Q*^2^, for $Q{R}_{g}\le 1$, where ${R}_{g}$ is the radius of gyration. Comparing these results to the plots of distribution of radii in Fig. 1, these are in the correct range. The ‘Dairy cream - 23’ had the largest droplet radius with the mean of the first peak about 2.5 µm, which is the reason for this being the only dairy sample with a Porod behaviour at the lowest *Q* value. For the ‘Soy milk - 11’, this sample showed a multimodal size distribution, where the mean of the second peak was 3.8 µm. These values of intensity weighted radius, dependent on the sixth power of the radius, are generally bigger than the values obtained from the surface area weighted radius obtained through Porod analysis. The reason for the slightly larger values obtained from the Porod analysis is the assumption of a sharp interface between the oil and water, which in this case has adsorbed emulsifier, creating a rougher surface and therefore an overestimate of the droplet radius. It is also possible, in the case of the plant-based materials, since not only oil droplets are present with dimensions in this size range, that other components contribute to surface area. The contrast of SLD is higher between protein or carbohydrate and water, which would affect the estimates of droplet radii.

Further analysis based on the intensity at the lowest value of *Q* provides information about the total fat content. In the low *Q* regime, the scattering intensity is primarily influenced by the concentration and size of the scattering objects as well as the scattering contrast between the dispersed material and the surrounding medium, $\triangle \rho$. The intensity scales with the square of the scattering contrast, and is linearly proportional to the volume fraction of scattered objects. The intensity also increases as the square of the droplet or particle volume (or the sixth power of the radius), indicating that larger droplets/aggregates with big scattering cross-sections significantly increase the scattering signal. With an SLD of oil similar for all samples, the two properties mainly affecting the intensity are the number of droplets and the size of the droplets. There are correlations between the intensity at a *Q* value of 0.0003 Å^−1^ and the total fat content. The polydispersity of these systems in combination with the presence of significant amounts of other materials with big dimensions, lead to slightly complex correlations. However, Spearman correlation coefficients for relationships between the intensity and the fat content were 0.56. With a null hypothesis of no correlation, the *p*-value was calculated as 0.002, suggesting that there is a significant correlation between intensity and total fat content. A plot of this is presented in Fig. 2e. This is useful for the investigation of other products, where the intensity at low *Q* is directly related to the fat content.

### Structural fingerprints

**Fig. 5:**
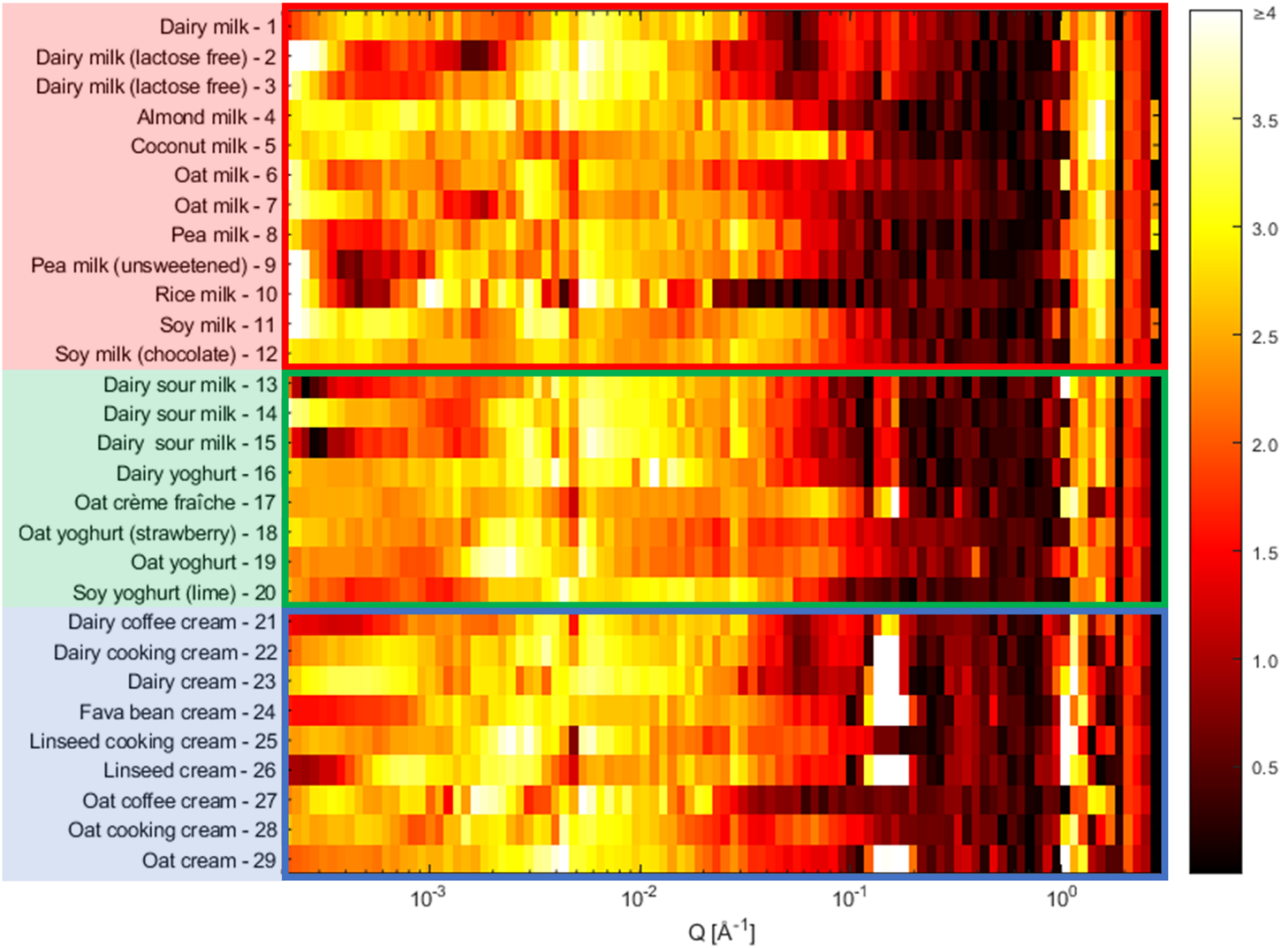
Fingerprint plots of scattered intensity from WAXS, SAXS and USAXS data. The data are presented as the absolute value of *d ln I/d ln Q* for intensity, *I*, and momentum transfer, *Q*. A linear temperature colour scale from 0 (black) to 4 (white) gradient is used, which is evaluated at 100 logarithmically distributed *Q* points. Colours represent milk (red), yoghurt (green) and cream (blue) products as described in the Materials section.

**Fig. 6:**
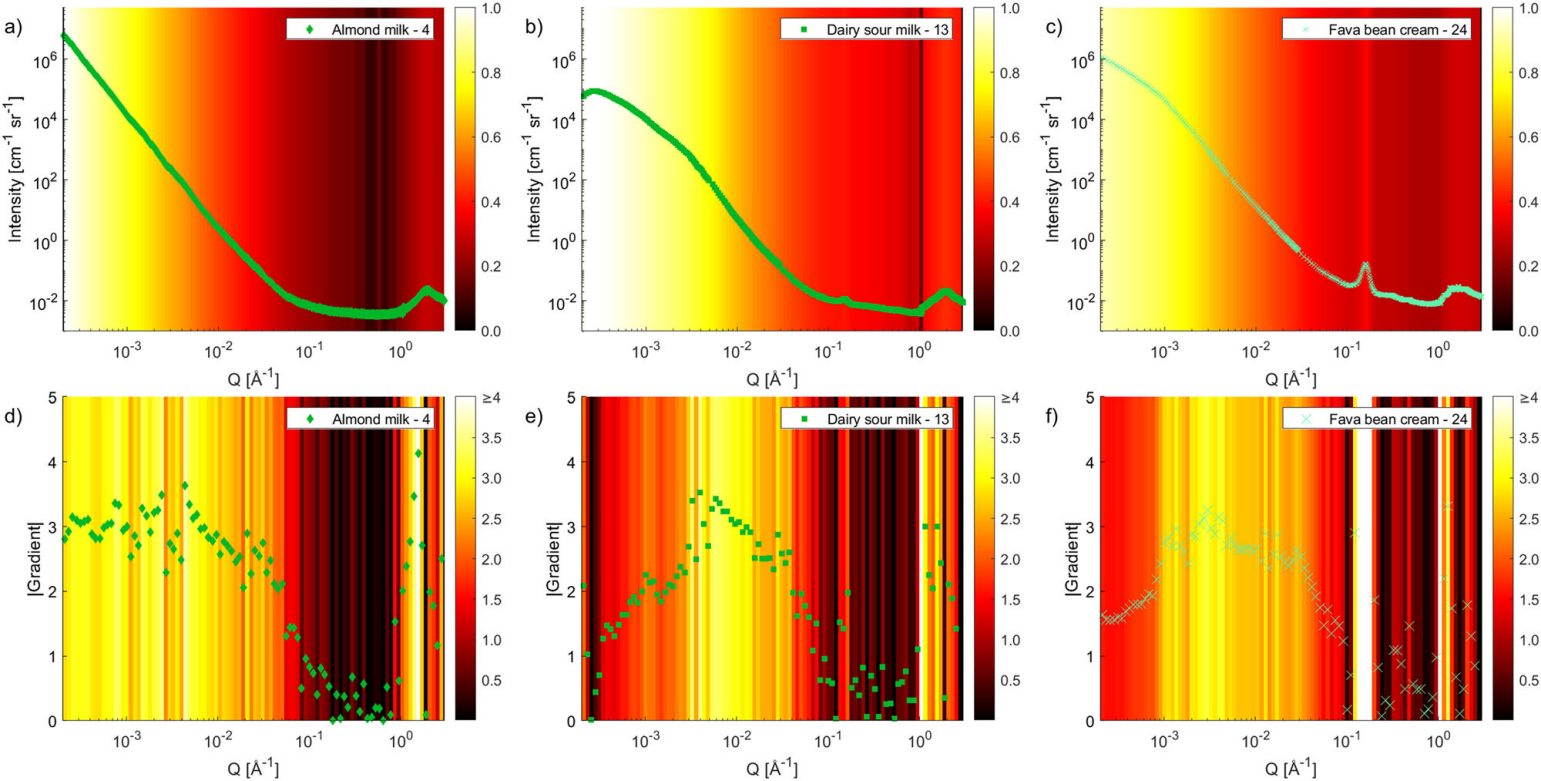
Fingerprint plots of scattered intensity and gradients from WAXS, SAXS and USAXS data for selected samples. In **a**, **d** ‘Almond milk - 4’, in **b**, **e** ‘Dairy sour milk - 13’, and in **c** and **f** ‘Fava bean cream - 24’. **a–c** Fingerprint plots of scattering data as normalised intensity, *I*, to the highest and lowest values for each sample with overlapping scattering pattern for the full *Q* range of (desmeared) USAXS, SAXS and WAXS data. **d**, **f** Fingerprint plots similar to the one described in Fig. 5 of the absolute value of *d ln I/d ln Q*, with overlapping gradient moduli curves for the full *Q* range of (desmeared) USAXS, SAXS and WAXS data.

To summarise the structural differences observed at multiple length scales for all samples in one plot, Fig. 5 presents a colour map of the gradients at different values of *Q* and identifies clearly the trends described in previous sections. These can be considered as the structural fingerprints of the various products. In this representation, the intensities have been linearly interpolated to 100 log distributed *Q* points, and the gradients have been calculated as *d ln I/d ln Q* between neighbouring points. The number of points was chosen based on the resolution of these measurements but should be evaluated based on the range of momentum transfer and instrument resolution as to avoid gradient variation of noisy points. The gradient moduli are plotted on a colour scale from dark ( | gradient | = 0) to light ( | gradient | ≥ 4). A brighter colour represents a steep decrease or increase, suggesting surface scattering (gradient ≈ −4), scattering from fractal structures (gradient ≈ −3 to −1) or correlation peaks (gradient ≈ > 1). The peak due to lamellar structure is clearly seen as the white band around 0.15 Å^−1^. Similarly, the correlation peaks in the WAXS region for the cream products as the lighter areas around 1–2 Å^−1^ are easy to observe. In general, the products in the milk category (red box) show fewer features, due to lack of crystalline and ordered structures. The bright colour at the lowest *Q* for these samples, suggests that there are big droplets and that their size is much greater than the inverse of the lowest value of *Q*. However, for some of the dairy products, a less steep gradient that decreases at the lowest *Q* (darker colour) indicates that predominantly smaller droplets are present. This structural fingerprint plot in Fig. 5 is helpful to provide an overview of the structures as it is easy to identify similarities and differences between the various categories and materials. It provides a representation on a universal scale, where materials can be added and excluded from the diagram without requiring a different colour scale. Rather than predicting exact structures of future products, this methodology can guide product development by comparing candidate formulations to existing structural fingerprints. In doing so, developers can aim to design materials with particular structural characteristics and, hence, physical properties similar to those of target products. Example fingerprint plots of the ‘Almond milk - 4’, ‘Dairy sour milk - 13’ and ‘Fava bean cream - 24’ samples are plotted in Fig. 6, where the scattering curves of desmeared USAXS data, merged to SAXS and WAXS data to cover the full *Q* range measured, have been added as overlays in the figure. There are clear differences in appearance of these samples. The almond milk (Fig. 6a, d) show clear surface scattering at low and intermediate *Q* values, with light colours without many features. The sample show a plateau (darker colours) starting below *Q* of 0.1 Å^−1^, due to the lower amount of carbohydrates in this sample. The sequence of bright, dark, bright regions for *Q* values greater than 1 Å^−1^ is the contribution from oxygen-oxygen spacing in water molecules observed for all samples. The dairy sour milk (Fig. 6b, e) has a more interesting behaviour in the low *Q* region, where darker colours indicate that the gradient of the scattering curve is starting to decrease, indicating smaller objects. In general, a slightly higher carbohydrate content contributes to some more scattering and less evidence of a plateau at intermediate *Q* values around 0.1 Å^−1^. The lamellae peak at 0.15 Å^−1^ is also clearly visible as a brighter band in this region. Small correlation peaks in the WAXS region contribute to the alternating dark/bright colours at large *Q* values compared to the almond milk sample. The fava bean cream (Fig. 6c, f) also shows a tendency to a low gradient at the smallest *Q* (orange colour), however this is not as pronounced as for the dairy sample. The orange colours of −3 to −2 gradient extend to higher *Q* values than the samples previously discussed, due to the high carbohydrate content. The lamellar structure peak due to the hydrogenated fats is clearly observed for this sample, where it is also easy to distinguish that the broadening is greater than for the dairy sample, indicating that there are more polydisperse structures in this plant-based sample. The multiple peaks of crystalline structures in the WAXS spectra are seen as alternating colours in this region. Fingerprint plots of this type of conventional scattering patterns combined with colour representation of the intensity as well as moduli of the gradients for all samples are presented in Figs. S13–S18 in the Supplementary Material. It is clear that this representation is very useful as it provides analysis of the shapes of the curves that make them readily accessible to a broad audience.

## Discussion

The structural findings for the plant-based alternatives to dairy products reported in this manuscript are useful and relevant in at least three ways. First, the broad selection of samples of different characters, products mimicking milk, yoghurt and cream, and the extensive collection of USAXS, SAXS and WAXS results, covering structures from Ångström to micrometre scales, adds to the existing base of data of scattering curves for food-grade emulsion systems. Secondly, correlating these patterns to specific properties of appearance, viscosity and texture is useful to link structural features to the physical properties perceived by the consumer. Although no direct relation to properties has been made here, readers are referred to, e.g., descriptions of how droplet size influence emulsion appearance and viscosity^43,44^. Finally, a focus on different ways to visualise scattering data is provided. A novel presentation of data as structural fingerprints, displaying both intensity and gradients in scattering curves, is proposed to facilitate intuitive interpretation. This lowers the barrier for engaging with scattering data, requires only a straightforward understanding of the underlying principles, and aims to support rather than replace expert analysis. It may also be a route to include AI tools more widely in data analysis. The further discussion and conclusions will be based on these three key points.

Scattering studies on plant-based emulsions are surprisingly rare, particularly for real food systems, even though the techniques cover many length scales and are commonly used to characterise other materials. Two of the main challenges with food emulsions are the presence of multiple components that adds to the complexity, and the lack of access to, and knowledge of, these techniques in the food science community. The present results can be viewed as a step towards establishment of a database of scattering results for a class of food systems. This can serve as a baseline to compare other products, materials and compositions and, for example, adds to the catalogue of different milk structures presented by de Kruif^45^. However, it is important to recognise that scattering data are highly dependent on instrumental design and data reduction protocols and therefore this should always be reported. The samples could also have very different structures dependent on their production, which would be an area for further separate investigation. However, with careful consideration, transparency and a broad selection of different types of samples, the results can be used both for the design of new formulations where a sample can be tuned to comply with a particular fingerprint, and for the prediction of formulations with desirable properties based on products with similar structures.

An eventual aim is to use the results to link structural features observed in the scattering patterns to physical properties such as rheology, mouthfeel and stability. There are challenges in applying scattering models directly to that goal but important trends and features are observed with model-independent correlations. Details regarding these types of correlations are shown in Fig. 2. Different ranges of momentum transfer give rise to scattered intensity that is correlated with total fat content, amount of solid fat content related to the saturation or hydrogenation of the fats, and carbohydrate concentration. A high carbohydrate content present in the aqueous phase for most of the plant-based products and particularly in the oat products, contributes to an increase in viscosity. A high fraction of solid fats derived from hydrogenated fats observed for the cream-type samples also contributes to a high viscosity. This is associated with ordered structures present at room temperature. The packing of the fat molecules is critical for the thermal stability, melting and mouthfeel experience, where the absence of β′ crystals for the plant-based products suggests that improvements to fully mimic the smooth texture of the dairy products might be feasible. The visual appearance of the various products seen in Fig. S2 in the Supplementary Material is affected by the emulsion droplet size that can be estimated from the DLS and USAXS data. A white appearance is generally preferred. The correlations could be exploited further in future studies such as investigating the response to thermal treatment, applied shear or the results of different preparation techniques.

A data presentation as fingerprint plots where different colours represent different intensities and gradients for scattering data was chosen as a means of presenting information in a compact form and as a method to facilitate easy interpretation. The gradients and intensities are the two main properties of a scattering curve that give information about the object size and shape, the contrast between different components, and the amount of various materials. With this representation, it is simple to distinguish ranges of momentum transfer where scattering proportional to specific surface areas (about −4 gradient or yellow on the temperature colour scale) or crystal structures (greater than |±4| gradient or white on the colour scale) are seen. Gradients of the order −3 to −1 are often characteristic of mass fractal structures^40^ and are the dominant behaviour of these samples over a large range of momentum transfer, *Q*. These observations are similar to previous detailed scattering studies of pea protein emulsions^42,46,47^ that identified fractal structures from individual particles to large clusters. The material necessary to cover an oil/water interface in most emulsions with large drops is far less than the total amount of vegetable biopolymers that are added. These do not act simply to modify oil/water interfacial energy but affect the viscosity and texture of these products. Using fingerprint plots, many samples can be compared simultaneously in a consistent and universal format. Instead of relating each sample to a baseline or average data set, presenting the gradient as well as the intensity of the data generalises the representation. We see strong potential in this approach, particularly for incorporating AI into data analysis, where the ability to compare large numbers of datasets systematically could provide valuable training data. It is particularly useful for such multi-component and complex systems where one single model is insufficient to describe a range of samples with different structures.

It is clear that the food products based on emulsions are both complex and diverse. However, substantial information is gained from combined USAXS, SAXS and WAXS measurements. We have demonstrated the potential of the techniques where each sample has a unique structural fingerprint that reflects its composition, production and structure. By correlating the structural features to different functional properties at multiple length scales, we can guide the development of formulations that are not only stable and tasty, but also healthier, for example reduced saturated fats, and more sustainable, for example choice of plant materials. While the structure of non-dairy systems differs from dairy emulsions, the fingerprint methodology enables a direct comparison by highlighting structural features that contribute to desirable product attributes. For example, information about fat crystal morphology and droplet size help to identify which plant-based systems can replicate key functional properties of dairy products, such as creaminess or mouthfeel. General features of the plant-based samples were broad scattering features characteristic of polydisperse samples and mixtures of different materials. The sizes of oil droplets and particles of other materials were generally bigger than those present in the dairy products. This was seen from the scattering dominated by the specific interfacial area (about −4 gradient) even at the lowest measured values of *Q*. This tendency is confirmed by the results of the dynamic light scattering measurements.

The structural differences observed between plant-based samples highlight the importance of ingredient selection (origin, extraction methods) and processing conditions (homogenisation, thermal treatment, processing) on product properties. This agrees with previous findings for emulsions stabilised with pea proteins^48^. The comparison between a broad selection of different oat-based materials demonstrates significant differences in structure that were larger than those observed for corresponding types of dairy products. The results suggest that plant-based substitutes offer flexibility in tuning properties to resemble dairy products. This study has contributed to deeper structural understanding of these materials. It has also identified efficient ways to investigate vegetable stabilized emulsions. The data allows simple comparison of structures of different classes of products prepared with materials of different origin.

## Methods

### Materials

**Fig. 7:**
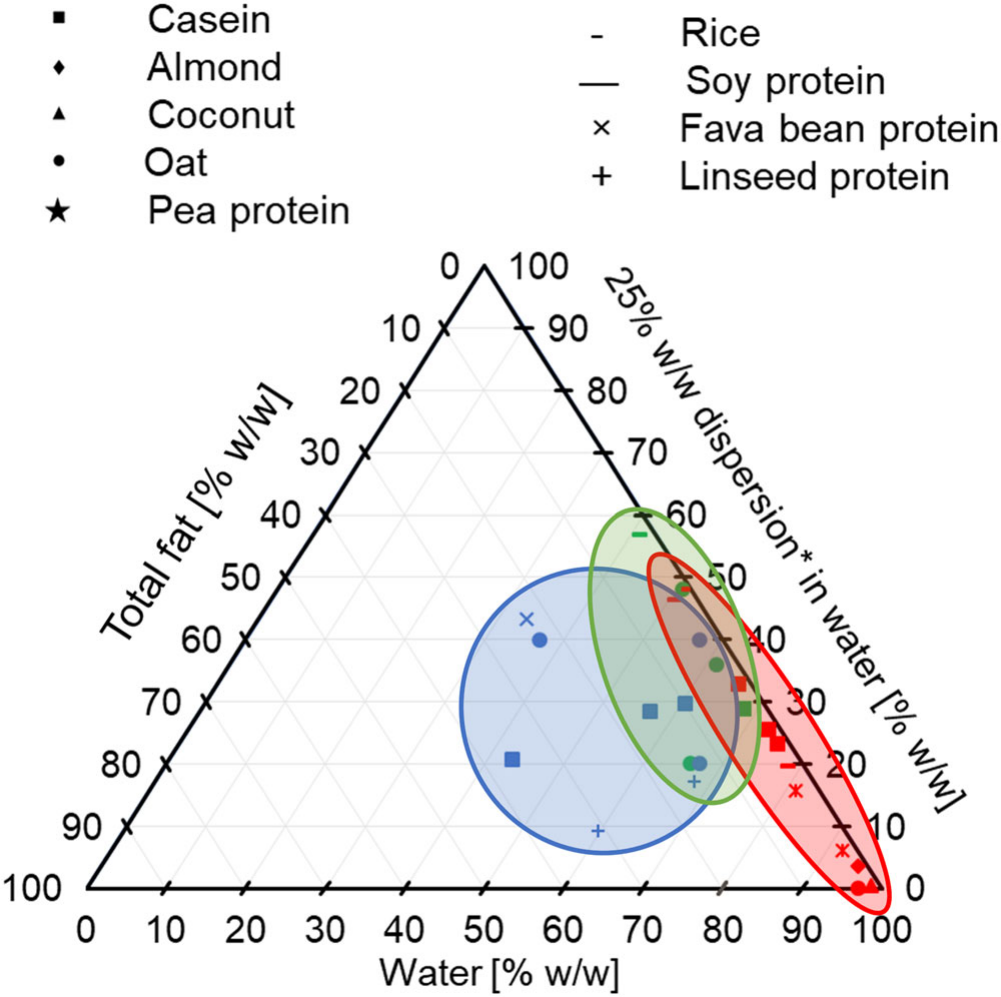
Ternary diagram featuring the compositions of the products investigated in this study. Different symbols correspond to different stabiliser material as labelled. Regions represent milk (red), yoghurt (green) and cream (blue) products as described in the Materials section. *dispersion of carbohydrate + protein materials.

Commercial dairy and plant-based milk products were obtained from local supermarkets in Uppsala, Sweden. The products include almond (diamond), casein (square), coconut (triangle), fava bean (cross), linseed (plus), oat (circle), pea (star), rice (short dash) and soy (long dash) based materials. A ternary diagram with the compositions is plotted in Fig. 7. In order to show compositions with low plant-material content better, the left corner represents 100% w/w total fat, the right corner represents 100% w/w water and the top corner corresponds to 100% w/w of a 25% w/w dispersion of the carbohydrate and protein materials in water. For example, the sample composition in the top corner is 0% fat, 25% w/w protein and carbohydrates, and 75% w/w water, whereas the centre point would have a sample composition of 33.3% w/w fat, 8.33% ( = 25/3) protein and carbohydrates, and 58.33% ( = 33.33 + 75/3) w/w water. The analysed samples were selected to represent a wide variety of materials and compositions. The materials have a fat content of 1.0–36%, carbohydrate from 0.0–13% and protein in the range 0.1–5.0%. The fat, carbohydrate and protein content, as well as the producer information are provided in Table 2. A fuller description of ingredients in the samples are provided in Table S3 in the Supplementary Material. For clarity, the samples were divided into three categories – “milk” (samples 1 to 12), “yoghurt” (samples 13 to 20) and “cream” (samples 21 to 29) products. This characterisation was based on composition and branding, where “milk” products have a low fat content ( ≤ 3% w/w) and are marketed for drinking purposes. “Cream” products are branded as whipping, cooking or coffee cream, and have a higher fat content ( ≥ 3% w/w fat). “Yoghurt” products were taken as all the fermented products. A European Union regulation for milk and milk products, restricts these words to the marketing of mammary secretion^49^ rather than other products. However, in this context they are used to describe the area of application of the plant-based alternatives as replacements for dairy milk/yoghurt/cream products. Throughout the plots in this paper, these categories are represented in colours of red for “milk” samples, green for “yoghurt” samples and blue for “cream” samples.

**Table 2:** Product compositions

Number	Sample	Total Fat^a^ [% w/w]	Carbohydrates [% w/w]	Protein [% w/w]	Trademark, Brand
1	Dairy milk	1.5	4.7	3.5	Roslagsmjölk AB
2	Dairy milk (lactose free)	1.5	2.4	3.4	Arla Ko, Arla Foods AB
3	Dairy milk (lactose free)	1.5	3.1	3.3	Valio Sverige AB
4	Almond milk	1.2	0.1	0.4	Garant, Axfood AB
5	Coconut milk	1.2	0.0	0.1	Alpro, Danone AB
6	Oat milk	3.0	7.1	1.1	Oatly AB
7	Oat milk	1.0	6.6	1.2	Oddly Good, Valio Sverige AB
8	Pea milk	3.0	1.9	2.0	Sproud International AB
9	Pea milk (unsweetened)	2.0	0.0	1.5	Sproud International AB
10	Rice milk	1.0	11	0.5	ICA, ICA Gruppen AB
11	Soy milk	2.0	0.8	3.5	Garant, Axfood AB
12	Soy milk (chocolate)	2.8	5.3	5.0	Alpro, Danone AB
13	Dairy sour milk	3.0	3.7	3.5	Arla Ko, Arla Foods AB
14	Dairy sour milk	3.0	3.7	3.5	Bollnäsfil, O. Kavli AB
15	Dairy sour milk	3.0	3.7	3.5	Coop, Coop Sverige AB
16	Dairy yoghurt	3.0	3.7	3.5	Arla Ko, Arla Foods AB
17	Oat crème fraîche	14	5.8	3.5	Oddly Good, Valio Sverige AB
18	Oat yoghurt (strawberry)	2.8	13	2.3	Arla JÖRĐ, Arla Foods AB
19	Oat yoghurt	1.1	12	1.5	Oatly AB
20	Soy yoghurt (lime)	2.0	10	3.6	Alpro, Danone AB
21	Dairy coffee cream	10	4.3	3.1	Kelda, Arla Foods AB
22	Dairy cooking cream	15	4.2	2.9	Kelda, Arla Foods AB
23	Dairy cream	36	2.9	2.3	Arla Köket, Arla Foods AB
24	Fava bean cream	23	10	0.8	Garant, Axfood AB
25	Linseed cooking cream	15	3.0	1.3	Flora, Flora Food Sweden AB
26	Linseed cream	31	1.7	0.6	Flora, Flora Food Sweden AB
27	Oat coffee cream	3.0	7.6	0.8	Garant, Axfood AB
28	Oat cooking cream	13	6.2	0.9	Oatly AB
29	Oat cream	23	11	0.5	Oatly AB

^a^The total fat is the oil or ‘fat’ content reported by the producer.

Samples 1 to 12 are milk products, samples 13 to 20 are yoghurt products and samples 21 to 29 are cream products as described in the Materials section

Samples were stored at 4 °C and allowed to equilibrate at room temperature, 22 °C, prior to all measurements. No additional processing or dilutions were applied to the samples, except when explicitly described under each measurement procedure.

### Dynamic light scattering (DLS)

The distributions of the hydrodynamic radii of the components in the various products were assessed by dynamic light scattering (DLS) with a Zetasizer Pro (Malvern Panalytical Ltd., Malvern, UK) instrument. A He-Ne laser with wavelength 633 nm was used and the measurements were done at 25 °C. The samples were diluted 1:100 to obtain a viscosity approximately that of water and to reduce multiple scattering. While for some systems dilution may affect the structure and droplet size^50^, it is useful to approximate ideas of the dimensions. The refractive index of the continuous phase was assumed to be that of water (1.33) and for the dispersed material it was set as 1.45. DTS1070 folded capillary cells were used for the measurements and each sample was measured in triplicate. The results are presented as volume-weighted distributions where error bars represent the range of values in repeated measurements. Radii distributions were derived using the Malvern adaptive algorithms to invert time correlation functions. The polydispersity index (PDI) is calculated as:

2 $${\rm{PDI}}=\frac{2{a}_{2}}{{a}_{1}^{2}}$$

where ${a}_{1}$ and ${a}_{2}$ are the first and second cumulants of simple fits to the time correlation function^51^. For a Gaussian distribution, this corresponds to ${{\rm{\sigma }}}^{2}/{{\rm{\mu }}}^{2}$, where ${\rm{\sigma }}$ is the standard deviation and ${\rm{\mu }}$ is the mean radius.

### Zeta potential

The zeta potential, ζ, was determined using the same Zetasizer Pro instrument as used for DLS with the same parameters for analysis. The zeta potential was calculated using the Malvern software based on Henry’s equation:

3 $${\rm{\zeta }}=\frac{3{U}_{E}{\rm{\eta }}}{2{\rm{\varepsilon }}f({\rm{\kappa }}{r}_{p})}$$

where ${U}_{E}$ is the electrophoretic mobility, ${\rm{\varepsilon }}$ is the dielectric constant and $f({\rm{\kappa }}{r}_{p})$ is Henry’s function. The Smoluchowski approximation for high ionic strength solutions of $f\left({\rm{\kappa }}{r}_{p}\right)=1.5$ was used. The results are presented in bar charts as average zeta potential for each sample with error bars of variability in repeated measurements.

### pH

The pH of the samples was measured using a FiveEasy pH Metre F20 (Mettler Toledo, Greisensee, Switzerland). The instrument was calibrated using buffer solutions of pH 4.00, 7.00 and 10.00. The measurements were performed at room temperature and are presented as the average of three repeated measurements of the same sample with uncertainty bars representing the range of measured values.

### X-ray scattering (USAXS, SAXS & WAXS)

X-ray scattering measurements were made with a Xeuss 2.0 Q-Xoom instrument (Xenocs, Grenoble, France) at Uppsala University. The instrument was equipped with a Cu K_α_ source with a wavelength of 1.54 Å and a Pilatus 3 R 300k detector (Dectris, Switzerland). Measurements were performed in vacuum at 22 °C in gel holders (2.5 × 2.5 mm^2^, spacer thickness 1 mm) sealed with Kapton windows. The actual thicknesses, due to the high viscosity of the samples and the flexibility of the Kapton as well as the need to ensure complete filling without bubbles, were in the range 1.5 ± 0.5 mm. The results are presented as plots of Intensity, *I*, versus momentum transfer, *Q,* defined in terms of angle, ${\rm{\theta }}$, and wavelength, ${\rm{\lambda }}$, as

4 $$Q=\left(\frac{4\pi }{{\rm{\lambda }}}\right)\sin \left(\frac{{\rm{\theta }}}{2}\right)$$

The experiments cover a broad *Q* range with overlapping points in different configurations, however USAXS, SAXS and WAXS data are plotted over the ranges of 0.0003–0.005, 0.005–1 and 1–3 Å^−1^, respectively. USAXS measurements were used to probe large scale structures of droplets and aggregates. SAXS results show nanoscale structures such as dispersed protein and carbohydrate structures. The WAXS technique measured smaller structures of crystalline molecular packing.

SAXS measurements with 2% instrument resolution were performed in two different configurations with sample-to-detector distances of 300 and 2400 mm. The acquisition time was 3600 s at each distance. WAXS data were collected simultaneously with a stationary detector Pilatus 100k (Dectris, Switzerland) at a distance of 161.2 mm. These data were plotted on an absolute scale of *I* by normalisation to the intensity of the transmitted direct beam and the sample thickness. The calibration was checked with glassy carbon^52^. Silver behenate as a sample with readily identified diffraction peaks was used to identify the detector position and hence calibrate *Q*. Noisy pixels were masked on the detector images and data reduction was made by azimuthal averaging of the 2D detector images to obtain 1D curves. The Kapton background scattering was subtracted from the sample scattering. The data were merged over the common data range for the three data sets (SAXS at 2400 mm, SAXS at 300 mm and WAXS). The data reduction was made using the XSACT software (Xenocs, Grenoble, France). For the USAXS measurements, the Bonse-Hart setup^53,54^ was used that has a slit length smearing of approximately 0.015 Å^−1^. These measurements took approximately 3 h and the data were reduced using the USAXSgui software (Xenocs, Grenoble, France). Desmearing of the USAXS data was made only for the fingerprint representation for the purpose of plotting it together with the SAXS data. For this purpose, the data were scaled and merged to the SAXS data over the range 0.0052–0.0055 Å^−1^. The fingerprint plots were created using custom Matlab code. These plots are presented in two different ways. First, the intensity is normalised to the highest and lowest value for each sample and plotted on a heat colour scale over the whole *Q* range at 100 logarithmically distributed points. Second, the modulus of the gradient of the intensity is plotted on a heat colour scale ranging from 0 to ≥ 4. The number of points in *Q* for the binning was chosen as a compromise between noise and resolution and should be evaluated for each study based on instrument resolution and the sample scattering.

## Supplementary information

SupplementaryMaterial

## Data Availability

Data and code for this article are available at Zenodo at 10.5281/zenodo.15020056.

## Abbreviations

DLS: Dynamic Light Scattering
PDI: Polydispersity Index
SAXS: Small-Angle X-ray Scattering
SLD: Scattering Length Density
USAXS: Ultra-Small-Angle X-ray Scattering
WAXS: Wide-Angle X-ray Scattering
