# Supplementary material for: From dairy to plant products: Understanding their structural fingerprints with X-rays
Source: NPJ Sci Food. 2025 Jun 23;9:109. doi: 10.1038/s41538-025-00493-w (PMC12185757; doi:10.1038/s41538-025-00493-w)
Supplement: Supplementary file 1 — SupplementaryMaterial [file 41538_2025_493_MOESM1_ESM.pdf]

Supplementary material

From dairy to plant products: Understanding their structural fingerprints with X-rays

Eleonora Olsmats* [a]

Adrian R. Rennie [a]

[a] Macromolecular Chemistry, Department of Chemistry – Ångström, Uppsala University, Box 538, 75121 Uppsala, Sweden

Eleonora.Olsmats@kemi.uu.se

Adrian.Rennie@kemi.uu.se

Table S1. Median values of radius from DLS measurements for samples plotted in Figure 1 in the main text. Samples 1 to 12 are milk products and samples 21 to 29 are cream products as described in the Materials section in the main text.

**Number**	**Sample**	**Peak position [Å]**
1	Dairy milk	1100
2	Dairy milk (lactose free)	1300
3	Dairy milk (lactose free)	1800
6	Oat milk	2100
7	Oat milk	500
8	Pea milk	2100
9	Pea milk (unsweetened)	1800
10	Rice milk	2100
11	Soy milk	3800
12	Soy milk (chocolate)	3800
21	Dairy coffee cream	3300
22	Dairy cooking cream	6000
23	Dairy cream	8200
24	Fava bean cream	5200
25	Linseed cooking cream	6000
26	Linseed cream	6000
28	Oat cooking cream	12900
29	Oat cream	3800

Table S2. Power law gradients of (desmeared) USAXS and SAXS data over the *Q* ranges 0.0003 to 0.001 Å^{-1} and 0.006 to 0.02 Å^{-1}, respectively. The samples with missing values did not have a constant power slope over the *Q* range chosen.

Number	Sample	Power law gradient between 0.0003 to 0.001 Å^{-1} (desmeared USAXS)	Power law gradient between 0.006 to 0.02 Å^{-1} (SAXS)
1	Dairy milk	3.35	3.62
2	Dairy milk (lactose free)	-	4.07
3	Dairy milk (lactose free)	-	3.85
4	Almond milk	3.80	3.41
5	Coconut milk	3.68	2.94
6	Oat milk	2.45	3.06
7	Oat milk	-	2.87
8	Pea milk	-	3.31
9	Pea milk (unsweetened)	-	3.27
10	Rice milk	-	3.51
11	Soy milk	3.89	2.74
12	Soy milk (chocolate)	3.47	2.90
13	Dairy sour milk	-	3.88
14	Dairy sour milk	-	3.89
15	Dairy sour milk	-	4.07
16	Dairy yoghurt	2.97	4.34
17	Oat crème fraîche	3.02	2.90
18	Oat yoghurt (strawberry)	3.00	2.80
19	Oat yoghurt	2.76	2.76
20	Soy yoghurt (lime)	2.20	3.66
21	Dairy coffee cream	-	3.34
22	Dairy cooking cream	3.19	3.62
23	Dairy cream	4.15	4.01
24	Fava bean cream	2.00	3.23
25	Linseed cooking cream	2.89	3.39
26	Linseed cream	-	2.97
27	Oat coffee cream	-	3.65
28	Oat cooking cream	3.14	3.41
29	Oat cream	2.68	3.48

Table S3. The ingredients list for each product is in order of decreasing content as specified by the manufacturer/producer. Samples 1 to 12 are milk products, samples 13 to 20 are yoghurt products and samples 21 to 29 are cream products as described in the Materials section in the main text.

**Number**	**Sample**	**Ingredients**	**Trademark, Brand**
1	Dairy milk	Low pasteurized milk, vitamin D	Roslagsmjölk AB
2	Dairy milk (lactose free)	High pasteurized milk, lactase enzyme, vitamin D	Arla Ko, Arla Foods AB
3	Dairy milk (lactose free)	High pasteurized milk, vitamin D, lactase enzyme, protease enzyme	Valio Sverige AB
4	Almond milk	Water, almond, calcium carbonate, salt, gellan gum, sunflower lecithin, guar gum, vitamin B2, vitamin B12, vitamin E, vitamin D	Garant, Axfood AB
5	Coconut milk	Water, coconut cream, coconut water, tricalcium phosphate, natural coconut flavouring, guar gum, xanthan gum, gellan gum, sea salt, vitamin B12, vitamin D2	Alpro, Danone AB
6	Oat milk	Water, oats, rapeseed oil, dipotassium phosphate, calcium carbonate, potassium iodide, salt, vitamin D2, vitamin B2, vitamin B12	Oatly AB
7	Oat milk	Water, gluten free oat meal, rapeseed oil, calcium, salt, iodide, vitamin B2, vitamin B12, vitamin D2	Oddly Good, Valio Sverige AB
8	Pea milk	Water, rapeseed oil, pea protein, agave syrup, dipotassium phosphates, calcium carbonate, calcium phosphates, lecithin, salt, vitamin B12, vitamin D, vitamin B2	Sproud International AB
9	Pea milk (unsweetened)	Water, pea protein, rapeseed oil, dipotassium phosphate, calcium carbonate, calcium phosphates, natural flavourings, lecithin, salt, vitamin B12, vitamin D, vitamin B2	Sproud International AB
10	Rice milk	Water, rice, sunflower oil, calcium carbonate, salt, vitamin D2, vitamin B12	ICA, ICA Gruppen AB
11	Soy milk	Water, soy beans, calcium carbonate, vitamin D, vitamin E, vitamin B2, vitamin B12	Garant, Axfood AB
12	Soy milk (chocolate)	Water, peeled soya beans, sugar, fat reduced cacao powder, calcium carbonate, flavouring, sea salt, potassium phosphates, gellan gum, vitamin B2, vitamin D2	Alpro, Danone AB
13	Dairy sour milk	High pasteurized milk, sour milk lactic fermenting agents, vitamin D	Arla Ko, Arla Foods AB
14	Dairy sour milk	High pasteurized milk, lactic acid fermenting agent, vitamin D	Bollnäsfil, O. Kavli AB
15	Dairy sour milk	High pasteurized milk, sour milk lactic fermenting agents, vitamin D	Coop, Coop Sverige AB
16	Dairy yoghurt	High pasteurized milk, lactic acid fermenting agent, vitamin D	Arla Ko, Arla Foods AB

Number	Sample	Ingredients	Trademark, Brand
17	Oat crème fraîche	Water, fully hydrogenated coconut oil, gluten free oat meal, fava bean protein, rapeseed oil, sugar, calcium, diacetyl tartaric acid ester of mono- and diglycerides, salt, vitamin B2, vitamin B12, vitamin D2, iodine, lactic fermenting agents, *Lactobacillus acidophilus*, *Bifidobacterium sp.*	Oddly Good, Valio Sverige AB
18	Oat yoghurt (strawberry)	Water, oats, strawberries, sugar, maize starch, rapeseed oil, fava bean protein, rice starch, maize fibre, agar, salt, beetroot juice concentrate, natural flavourings, pectin, natural strawberry flavouring, lemon juice concentrate, calcium citrates, vitamin D, vitamin B9, cultures (*Streptococcus thermophilus*, *Lactobacillus delbrueckii subsp. Bulgaricus*)	Arla JÖRÐ, Arla Foods AB
19	Oat yoghurt	Water, oat, potato starch, rapeseed oil, modified potato starch, potato protein, calcium phosphate, calcium carbonate, potassium iodine, apple acid, lactic acid, salt, vitamin D2, vitamin B2, vitamin B12	Oatly AB
20	Soy yoghurt (lime)	Water, peeled soya beans, sugar, glucose fructose syrup, lemon juice from concentrate, lemon, lime juice from concentrate, citric acid, sodium citrates, pectin, tricalcium phosphate, sea salt, carrot concentrate, pumpkin concentrate, tocopherol-rich extracts, fatty acid esters of ascorbic acid, natural lemon flavouring, other natural flavourings, vitamin B2, vitamin B12, vitamin D2, yoghurt lactic fermenting agents (*Streptococcus thermophilus*, *Lactobacillus bulgaricus*)	Alpro, Danone AB
21	Dairy coffee cream	Cream	Kelda, Arla Foods AB
22	Dairy cooking cream	Cream, carrageenan	Kelda, Arla Foods AB
23	Dairy cream	Pasteurized cream, carrageenan	Arla Köket, Arla Foods AB
24	Fava bean cream	Fully hydrogenated coconut fat, fava bean, fully hydrogenated rapeseed fat, dextrose, sucrose, salt, diacetyl tartaric acid ester of mono and diglycerides, polyoxyethylene sorbitan monostearate (Tween 60), trisodium citrate, calcium carbonate, hydroxypropyl methylcellulose	Garant, Axfood AB
25	Linseed cooking cream	Water, rapeseed oil, coconut fat, linseed protein, modified potato starch, polysorbate 60, methyl cellulose, xanthan gum, trisodium citrates, natural flavourings, salt, beta carotene	Flora, Flora Food Sweden AB
26	Linseed cream	Water, coconut fat, rapeseed oil, linseed protein, sugar, modified corn starch, sunflower lecithin, sugar ester of mono and diglycerides of fat acids, guar gum, carob flour, natural flavourings, salt, beta carotene	Flora, Flora Food Sweden AB

Number	Sample	Ingredients	Trademark, Brand
27	Oat coffee cream	Water, oat, rapeseed oil, potassium phosphates, calcium carbonate, salt, vitamin B2, vitamin D2, vitamin B12	Garant, Axfood AB
28	Oat cooking cream	Water, rapeseed oil, oat, diacetyl tartaric acid ester of mono- and diglycerides, xanthan gum, salt	Oatly AB
29	Oat cream	Water, fully hydrogenated coconut oil, fully hydrogenated rapeseed oil, oat, maltodextrin, diacetyl tartaric acid ester of mono- and diglycerides, polyoxyethylene sorbitan monostearate (Tween 60), salt, trisodium citrate, hydroxypropyl methylcellulose, gellan gum, beta carotene	Oatly AB

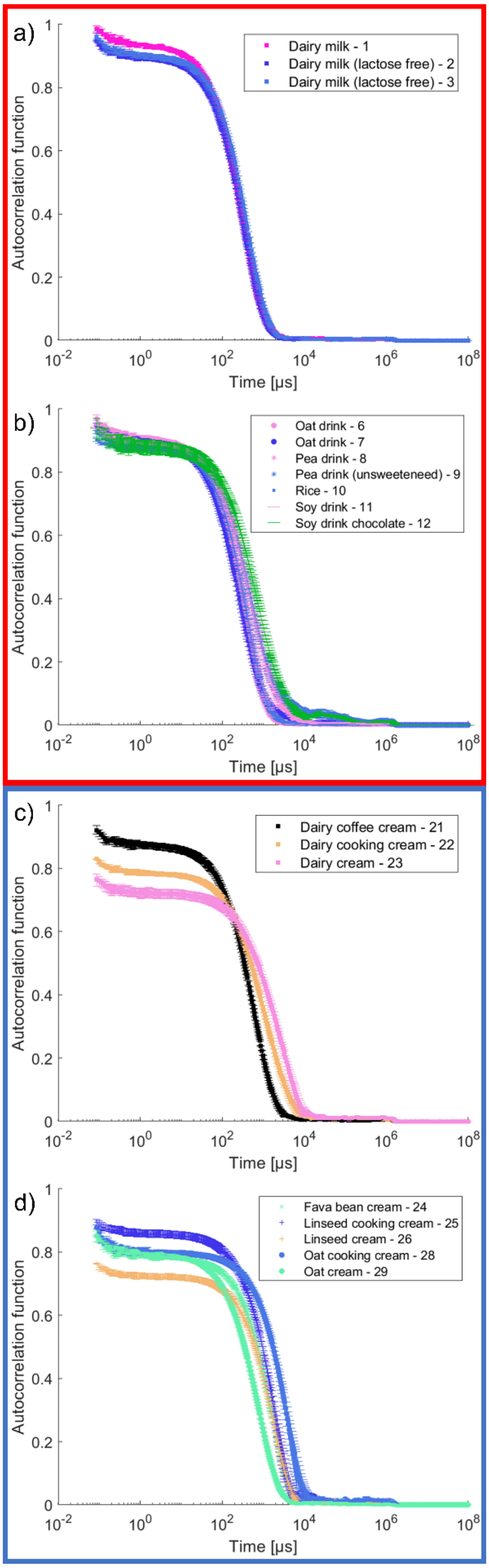

Figure S1. Time correlation functions from DLS for the data presented in Figure 1 in the main text. a) conventional dairy milk, b) plant-based milk, c) conventional dairy cream, and d) plant-based cream products. Error bars show variation for measurements performed in triplicate.

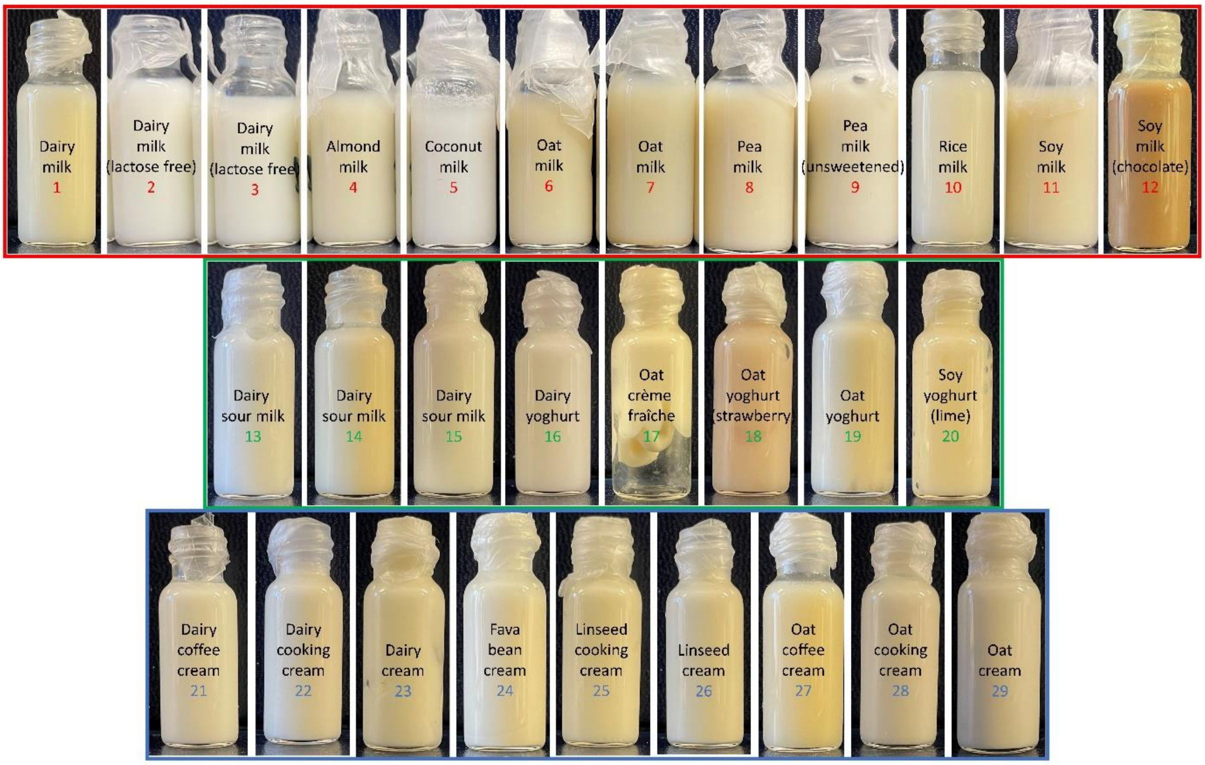

Figure S2. Photographs showing the visual appearance of products used in this study. Colours represent milk (red), yoghurt (green) and cream (blue) products as described in the Materials section in the main text.

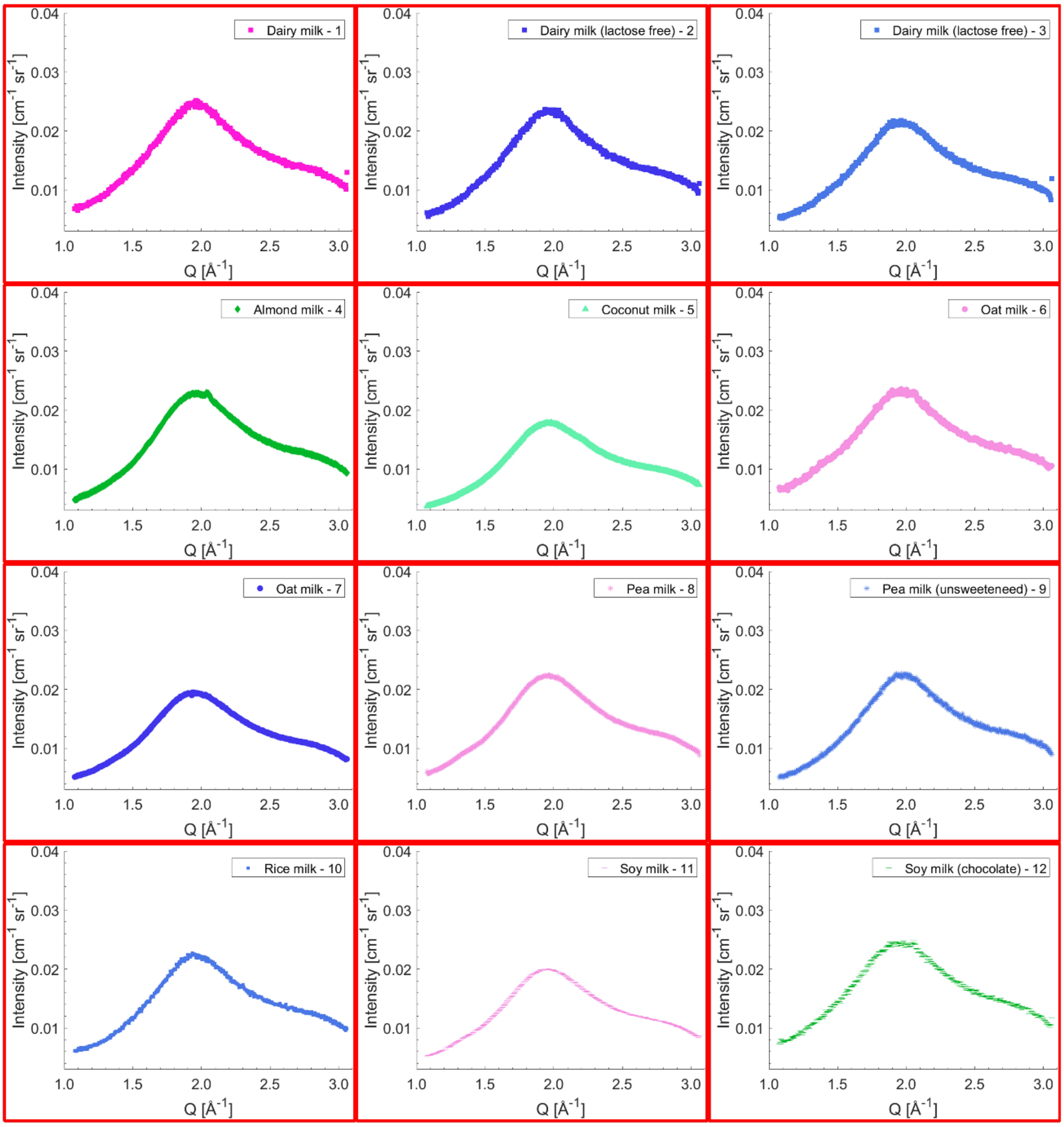

Figure S3. WAXS data for the milk products used in this study.

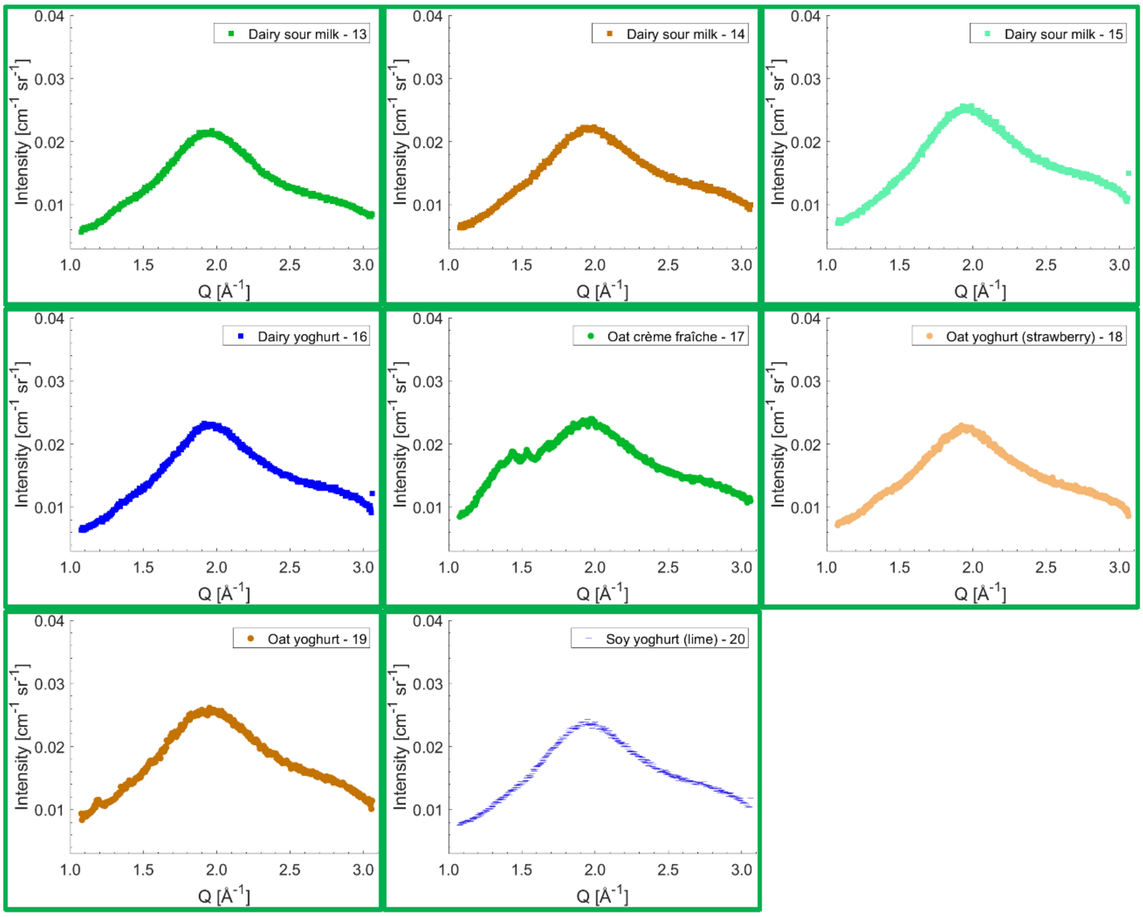

Figure S4. WAXS data for the yoghurt products used in this study.

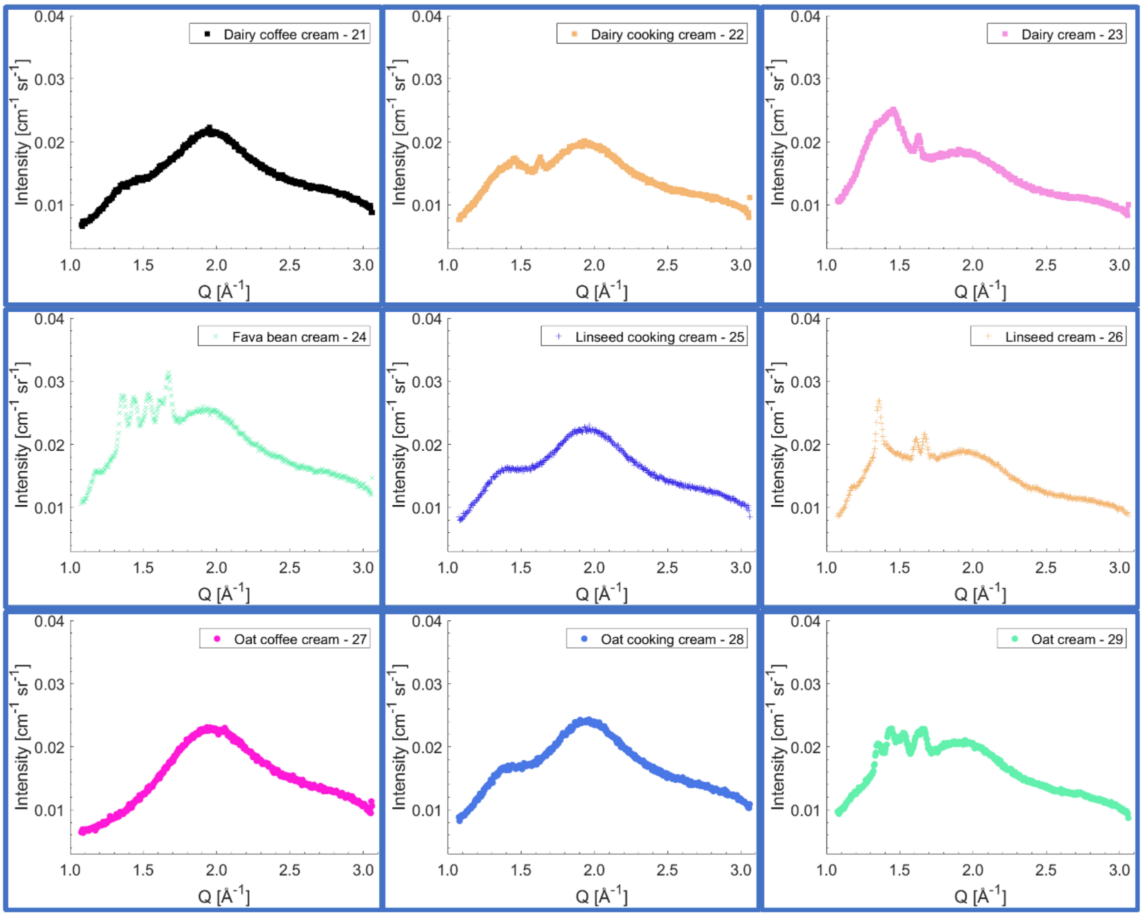

Figure S5. WAXS data for the cream products used in this study.

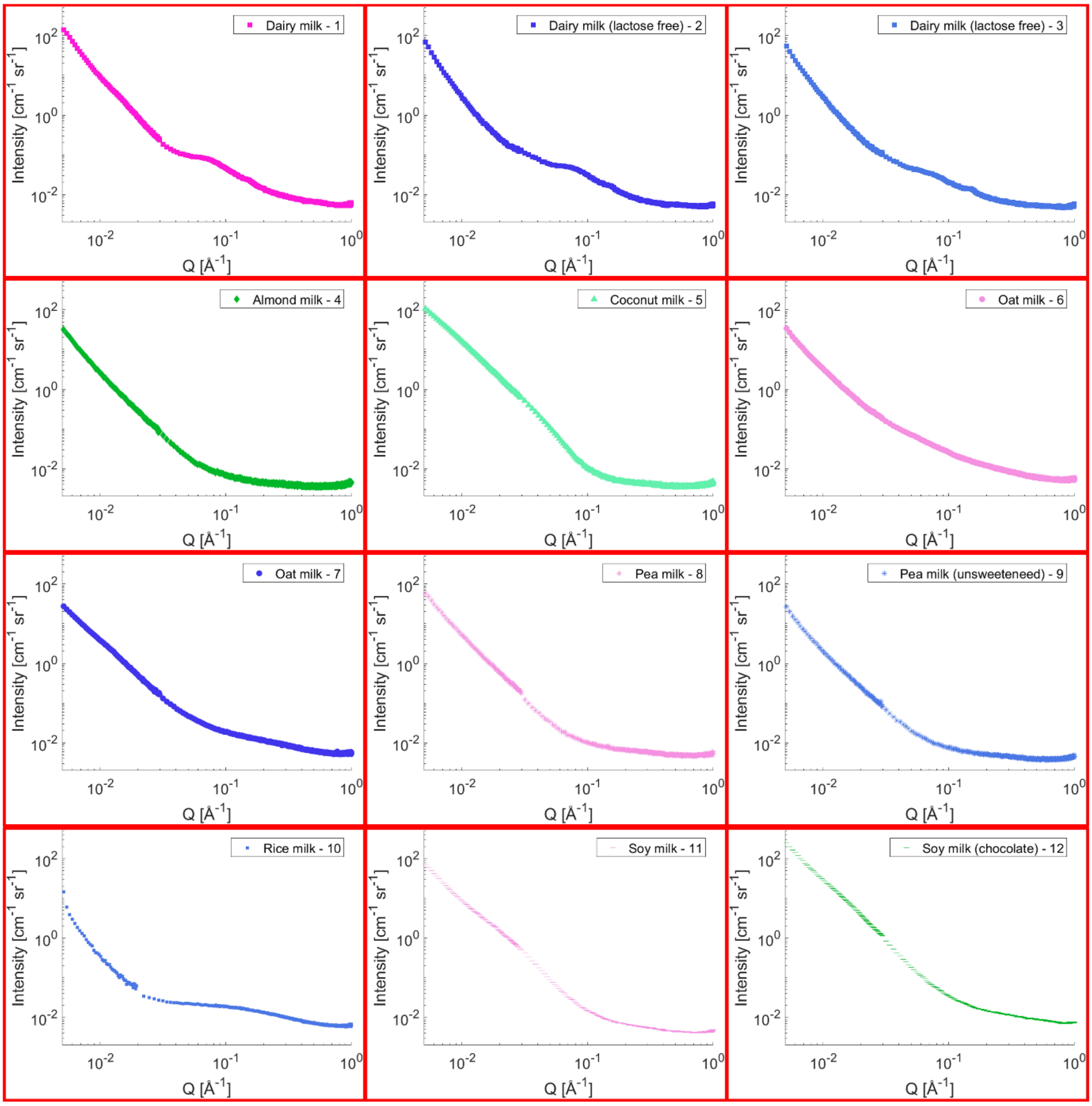

Figure S6. SAXS data for milk products used in this study.

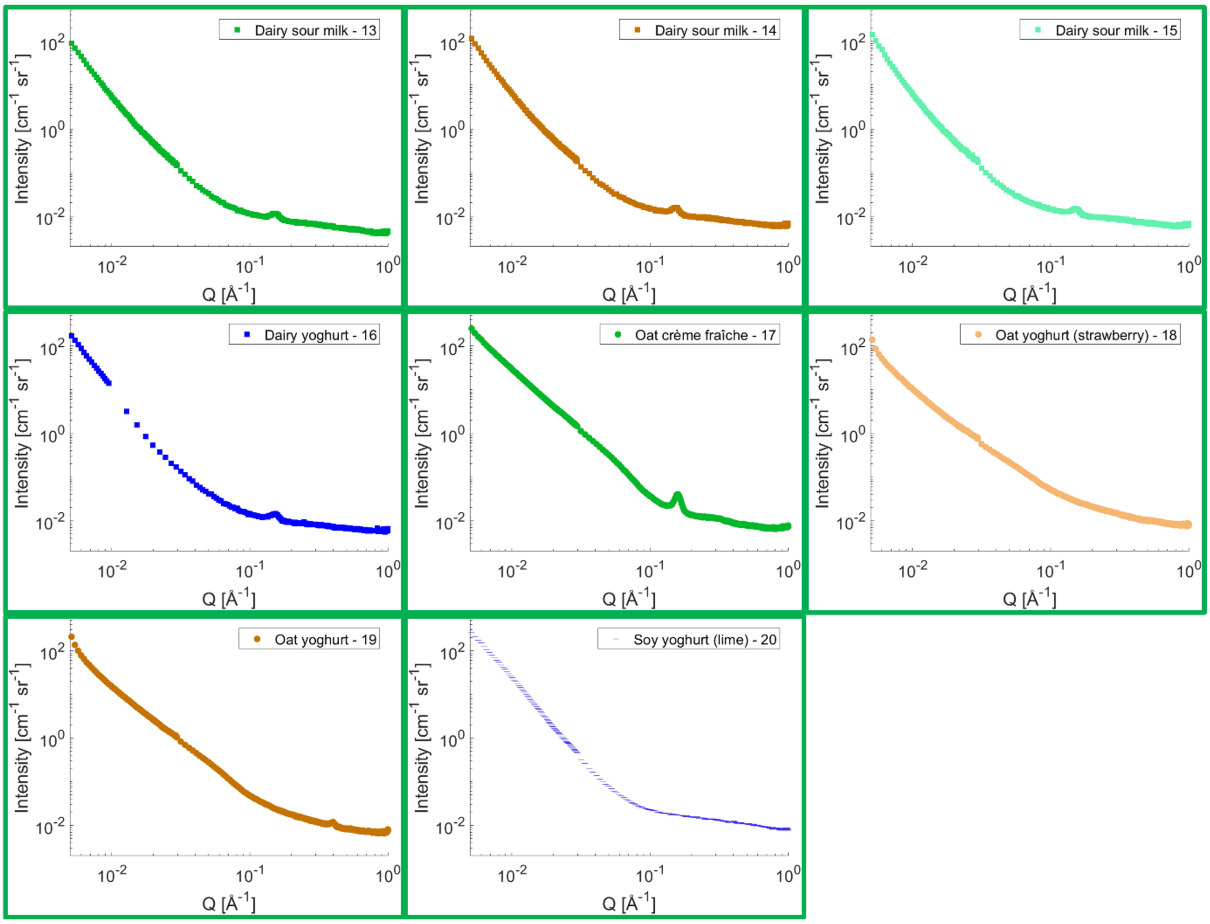

Figure S7. SAXS data for yoghurt products used in this study.

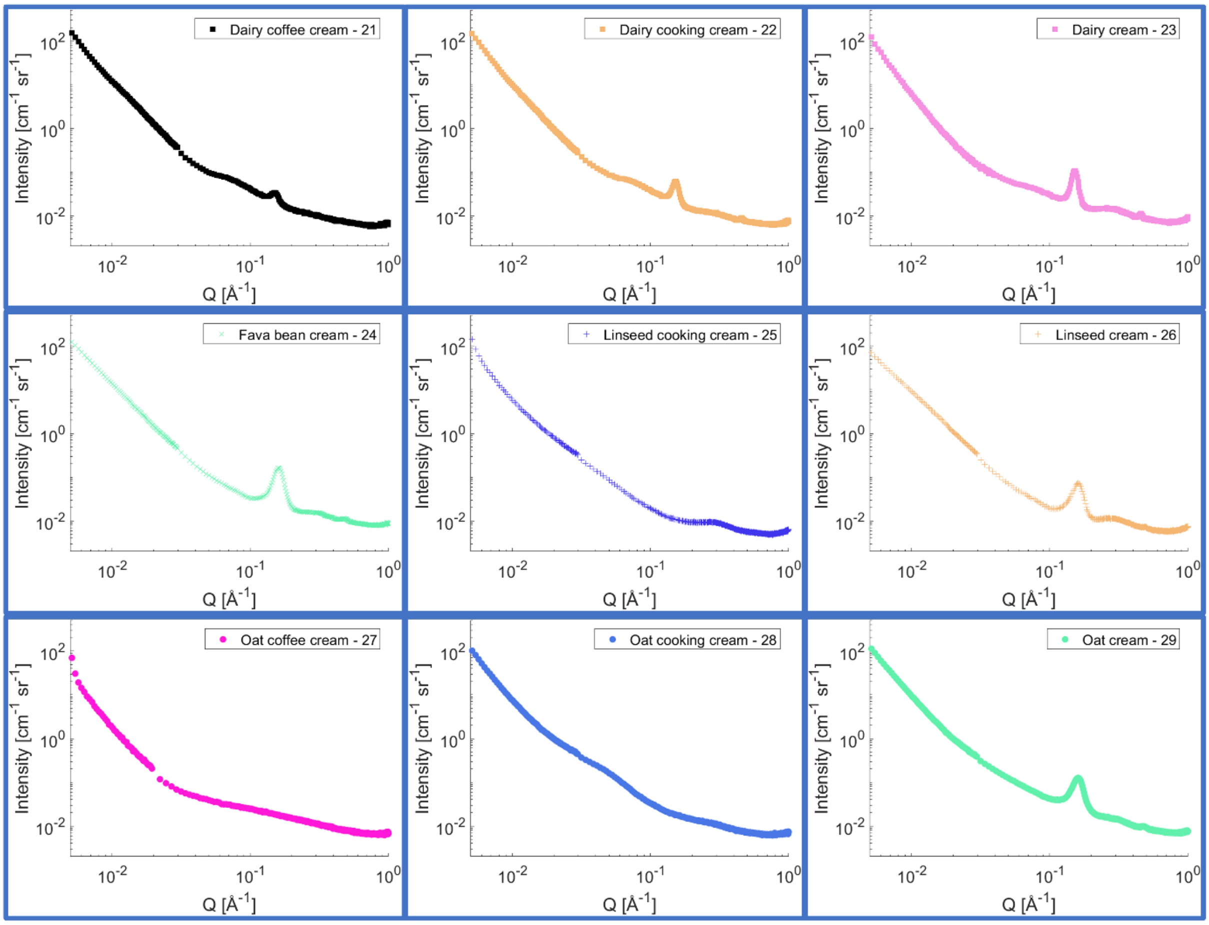

Figure S8. SAXS data for cream products used in this study.

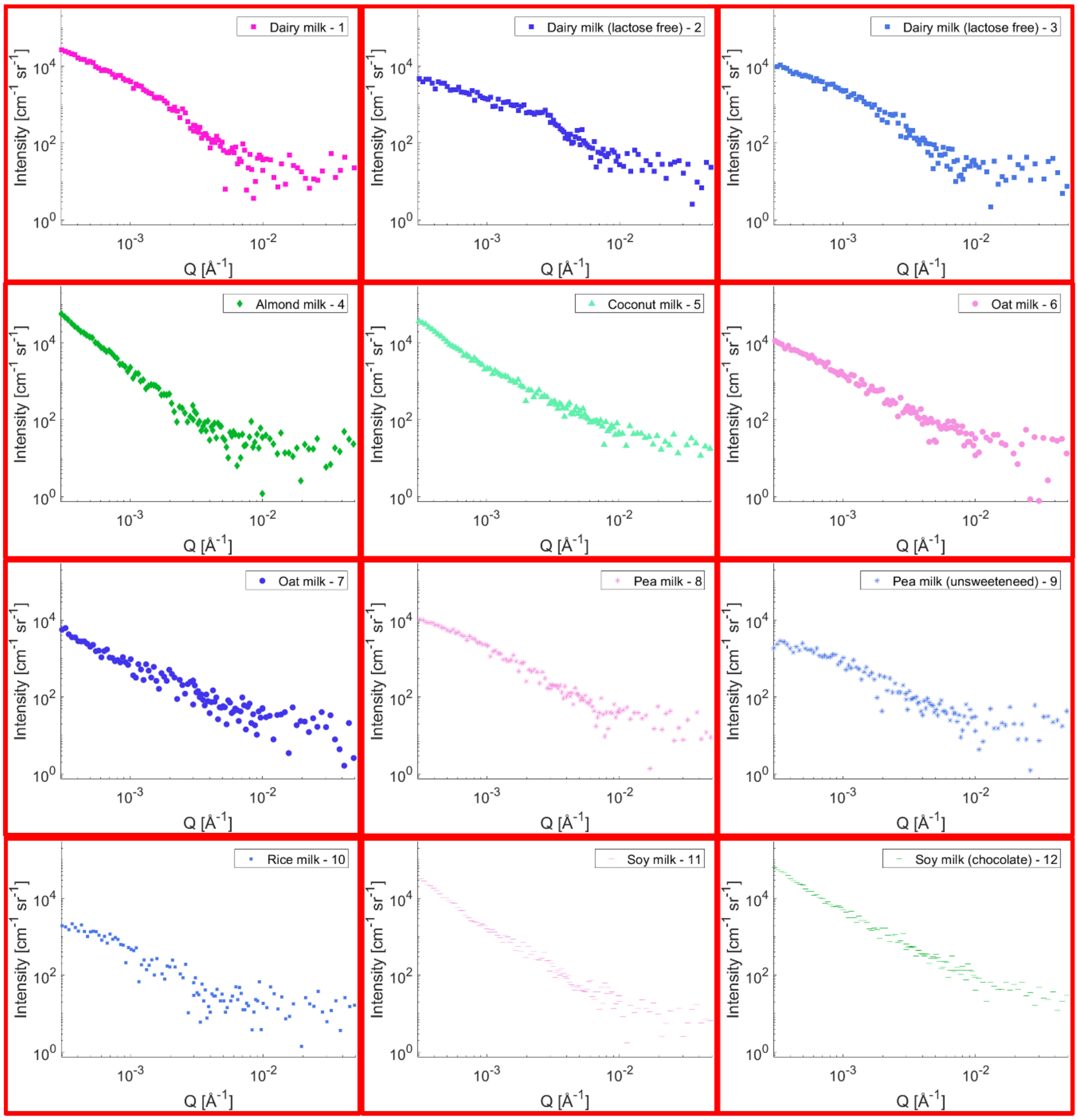

Figure S9. USAXS data for milk products used in this study.

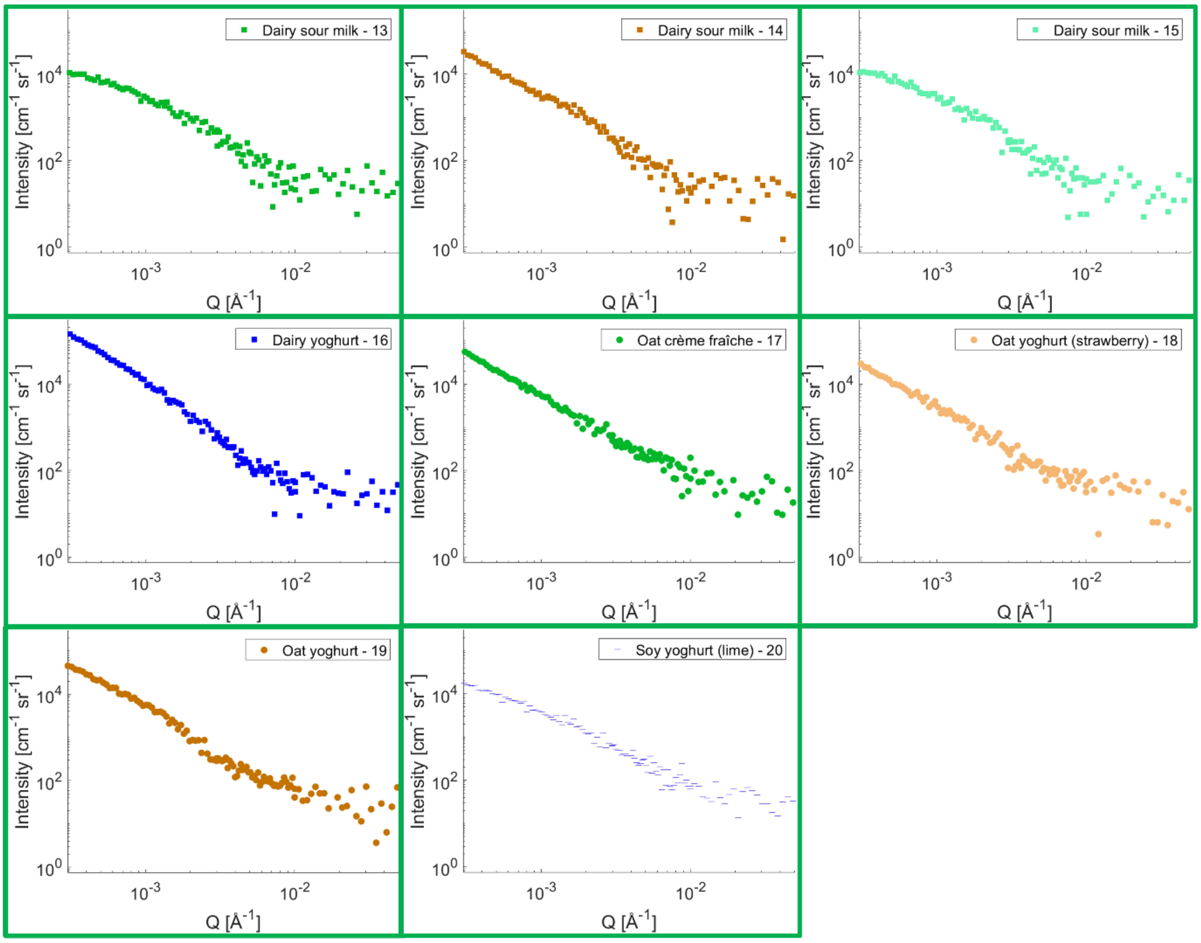

Figure S10. USAXS data for yoghurt products used in this study.

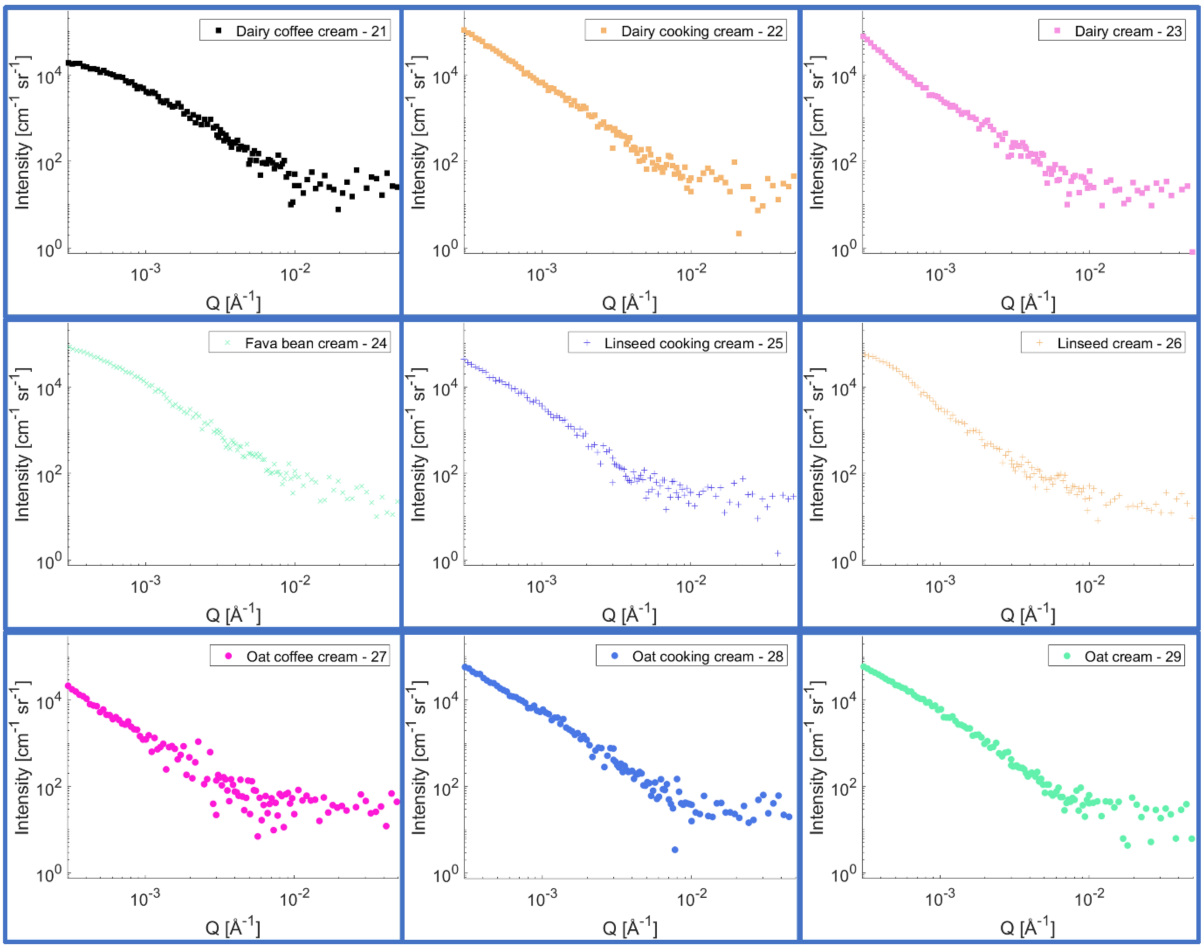

Figure S11. USAXS data for cream products used in this study.

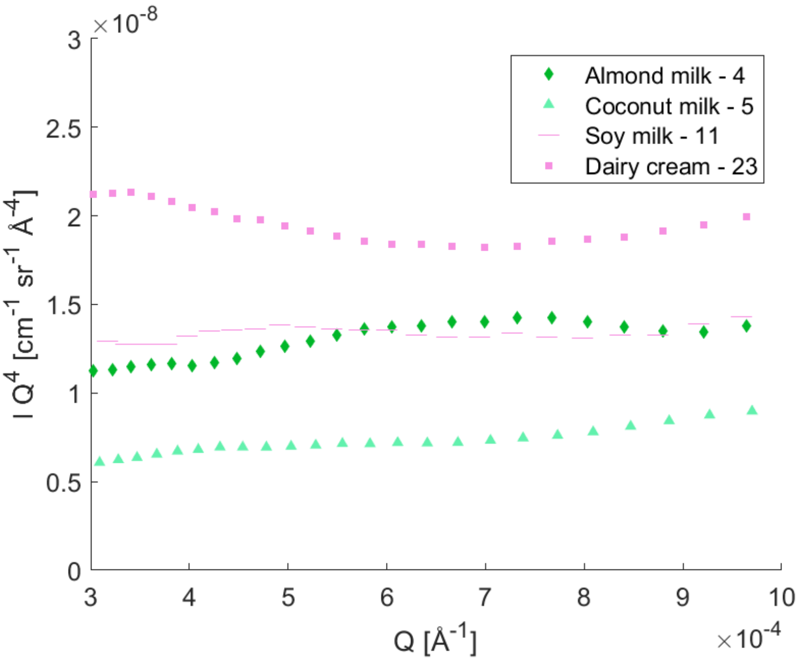

Figure S12. Porod plot of IQ^4 versus Q for selected samples. Samples that show a constant value at the lowest Q values are 'Almond milk – 4',' Coconut milk – 5', 'Soy milk – 11' and 'Dairy cream – 23'. These values can be used to estimate the specific surface area as described by Equation 1 in the main text.

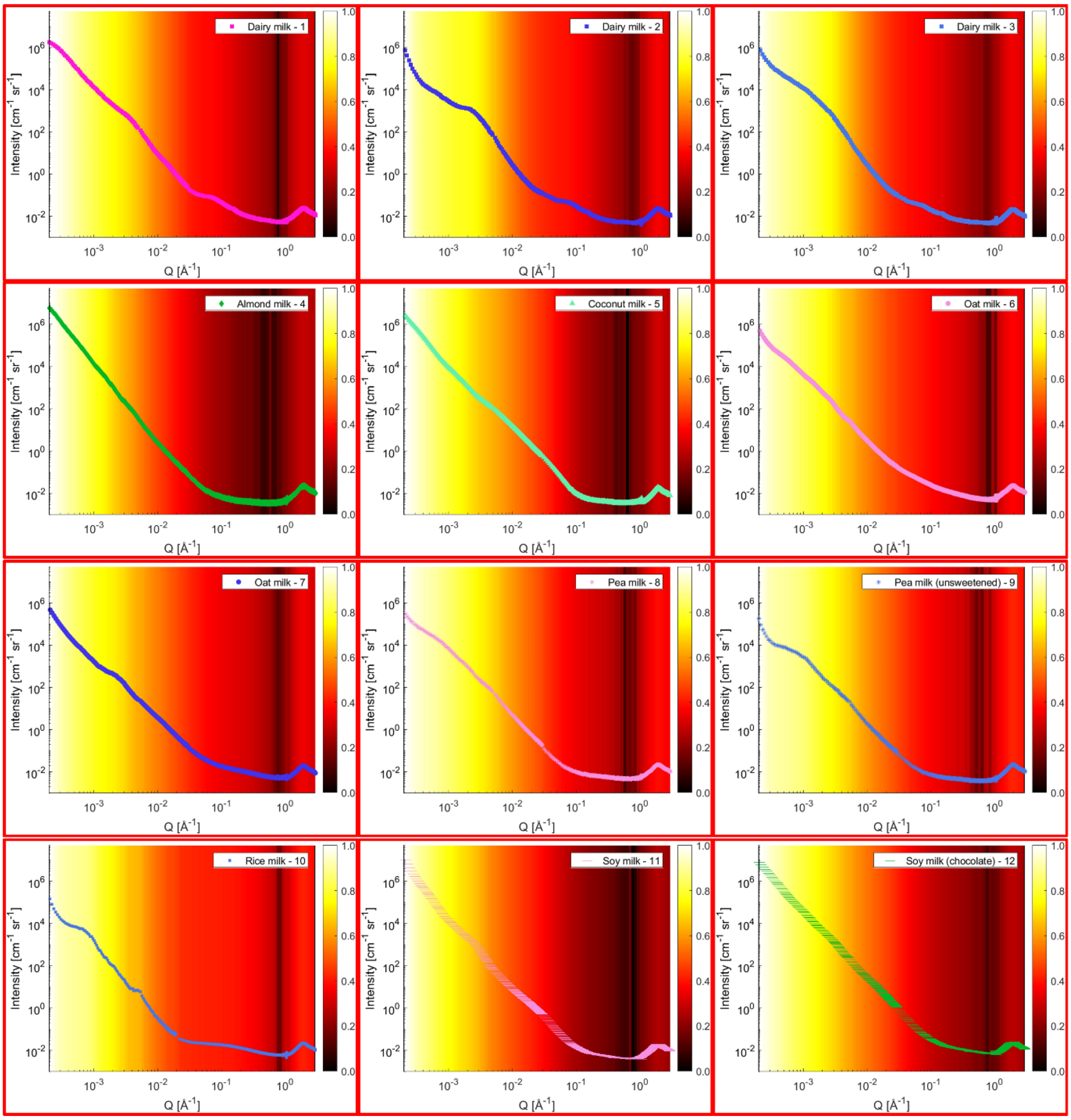

Figure S13. Structural fingerprints and USAXS, SAXS and WAXS scattering patterns for milk products used in this study. Plots show normalised intensity, *I*, to the highest and lowest values for each sample with overlapping scattering pattern for the full *Q* range of (desmeared) USAXS, SAXS and WAXS data.

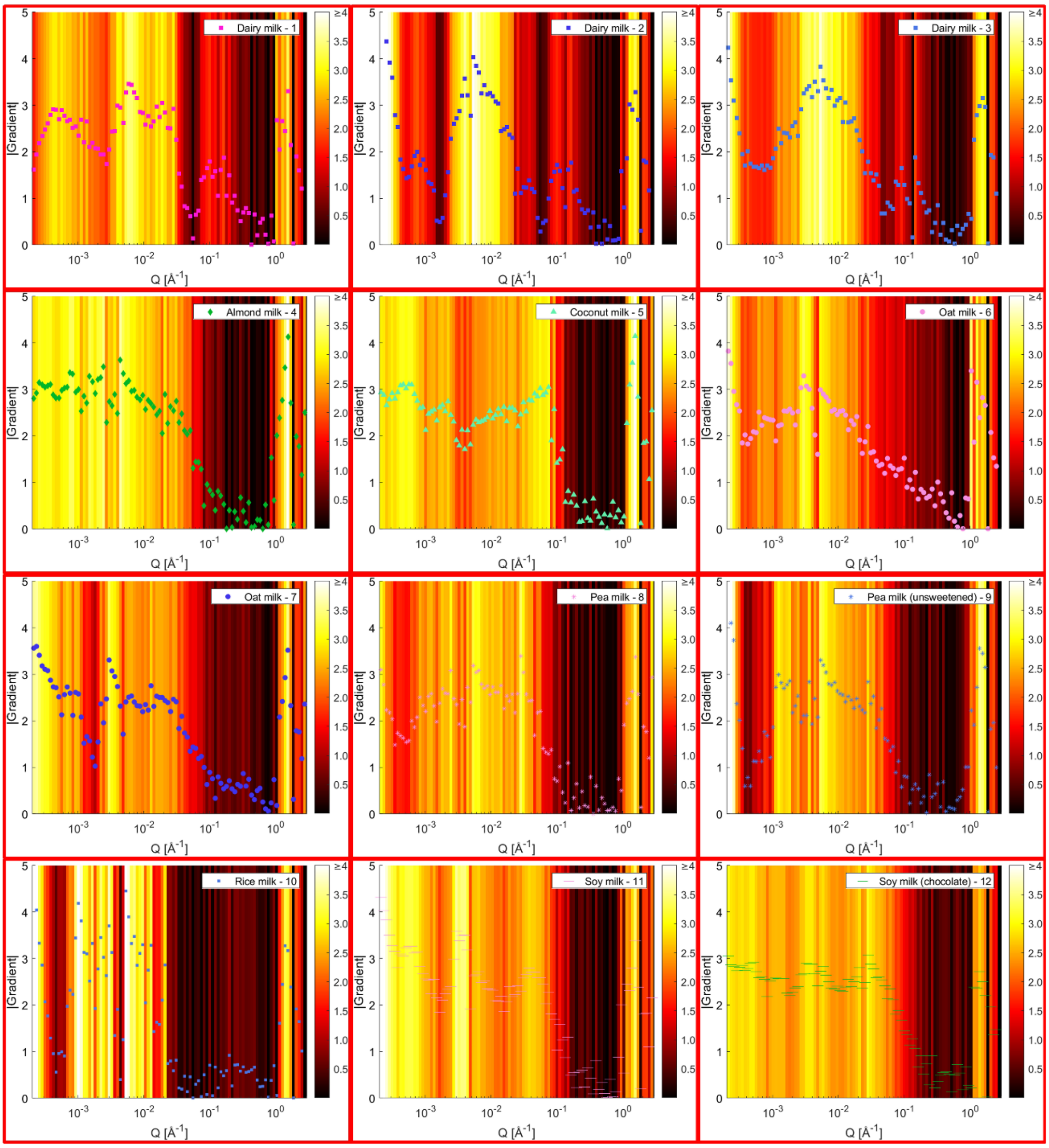

Figure S14. Structural fingerprints of the absolute value of *d ln I/d ln Q* for milk products used in this study, with overlapping slope moduli curves for the full *Q* range of (desmeared) USAXS, SAXS and WAXS data.

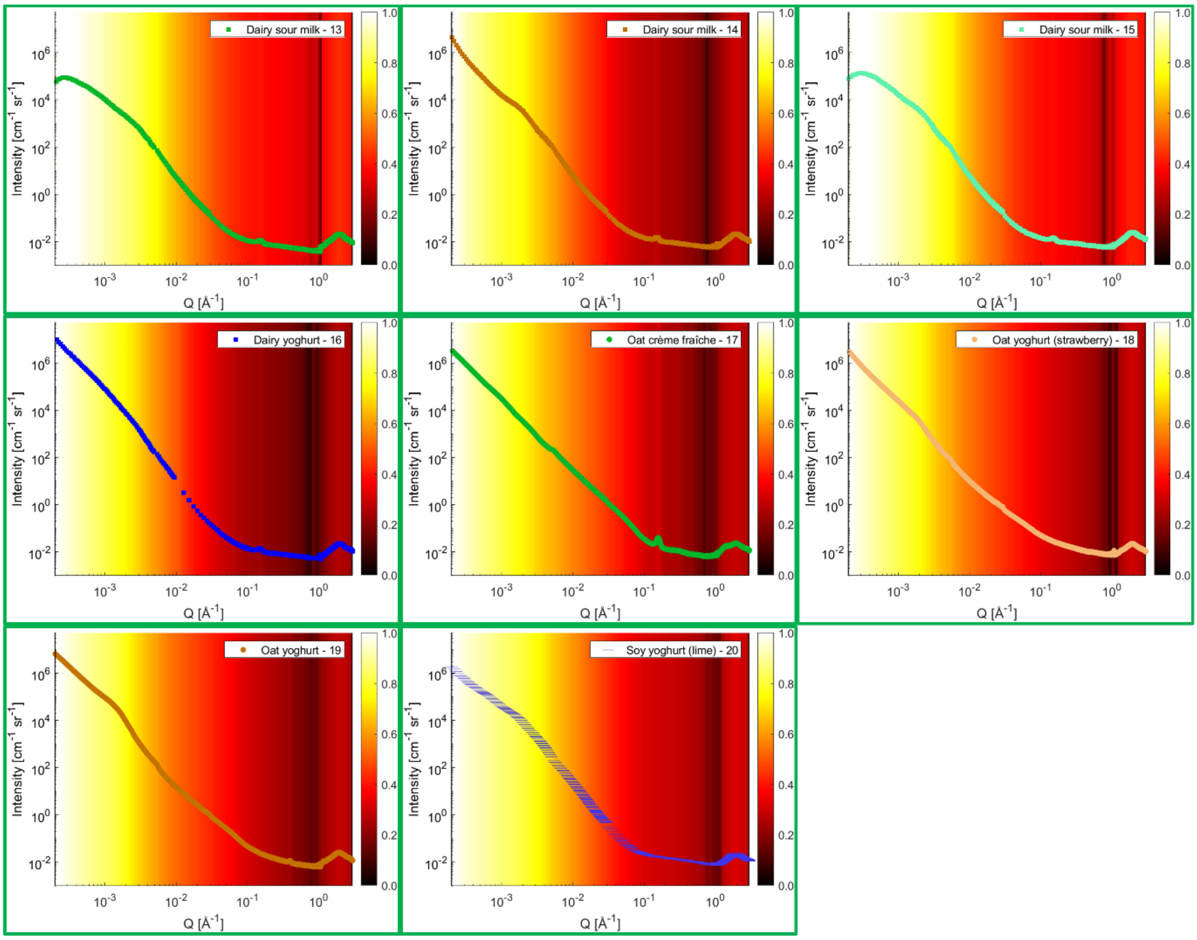

Figure S15. Structural fingerprints and USAXS, SAXS and WAXS scattering patterns of yoghurt products used in this study. Plots show normalised intensity, *I*, to the highest and lowest values for each sample with overlapping scattering pattern for the full *Q* range of (desmeared) USAXS, SAXS and WAXS data.

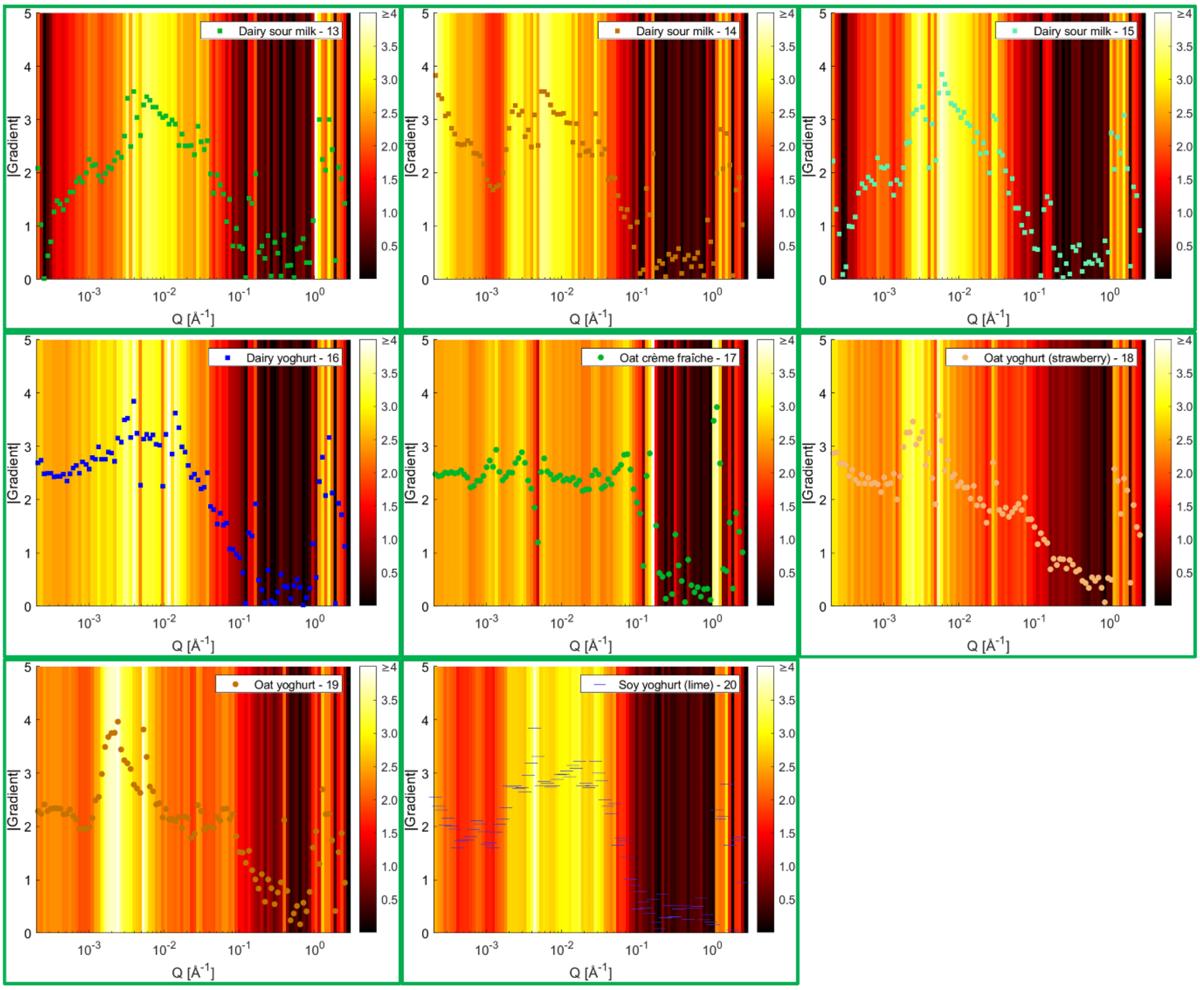

Figure S16. Structural fingerprints of the absolute value of *d ln I/d ln Q* for yoghurt products used in this study, with overlapping slope moduli curves for the full *Q* range of (desmeared) USAXS, SAXS and WAXS data.

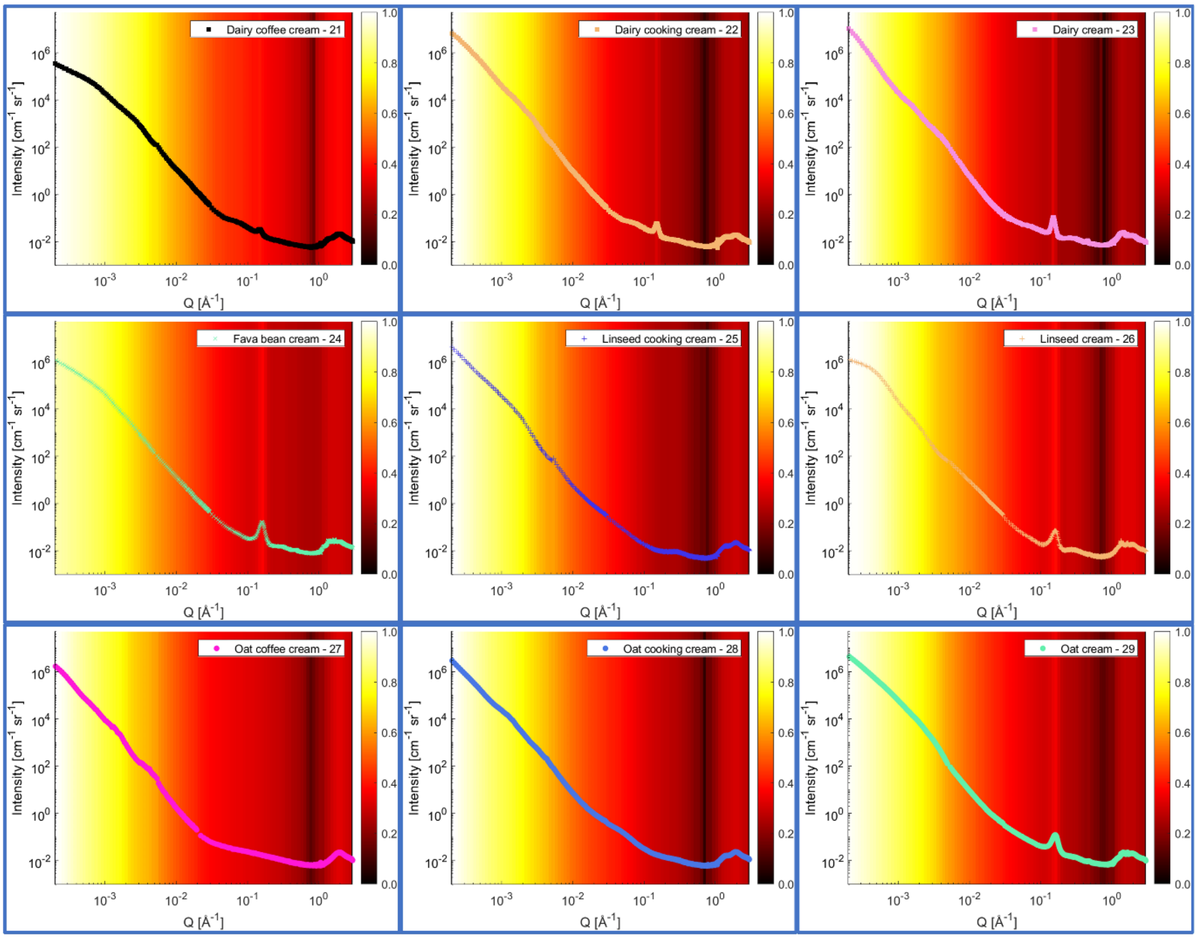

Figure S17. Structural fingerprints and USAXS, SAXS and WAXS scattering patterns for cream products used in this study. Plots show normalised intensity, *I*, to the highest and lowest values for each sample with overlapping scattering pattern for the full *Q* range of (desmeared) USAXS, SAXS and WAXS data.

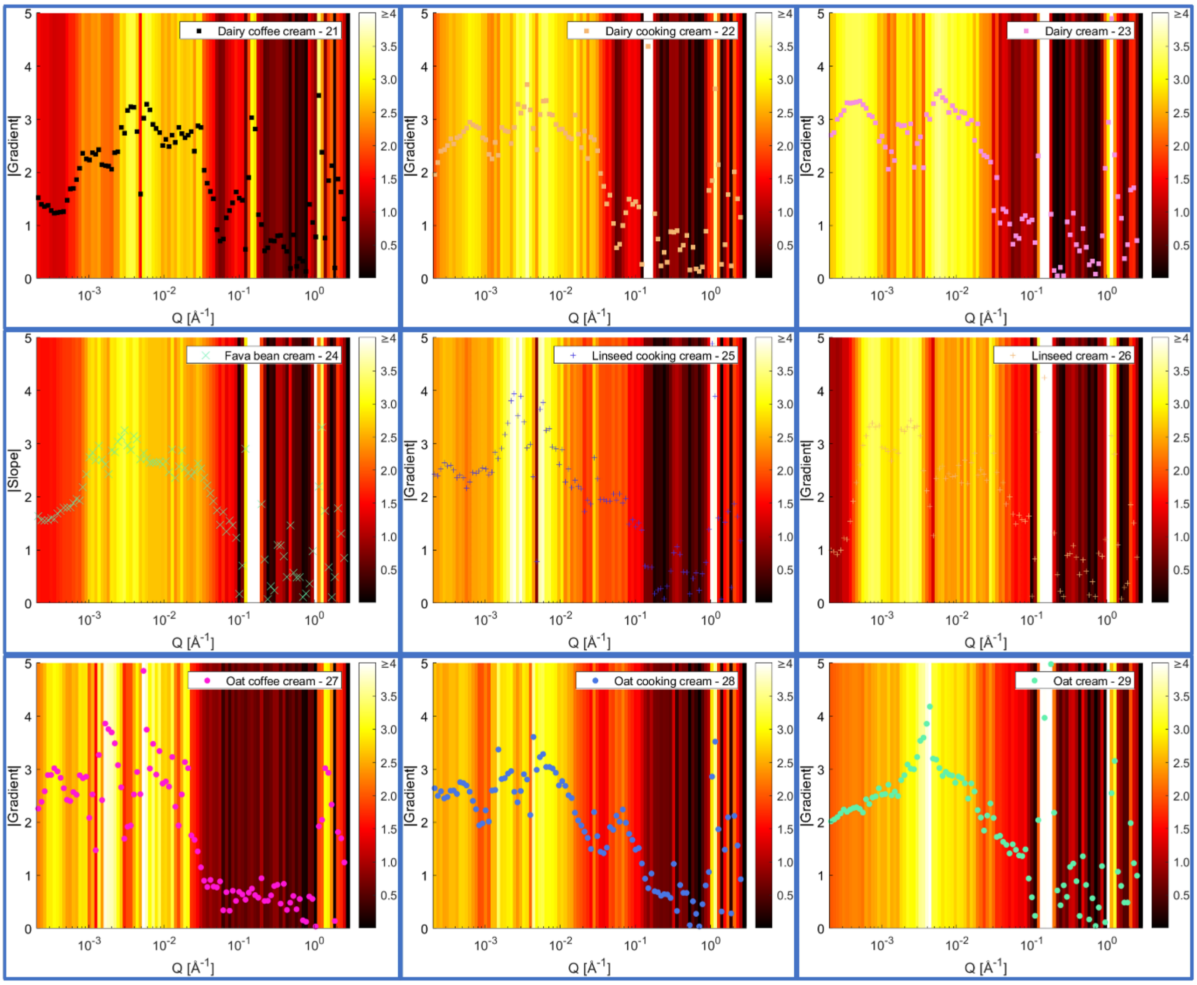

Figure S18. Structural fingerprints of the absolute value of *d ln I/d ln Q* for cream products used in this study, with overlapping slope moduli curves for the full *Q* range of (desmeared) USAXS, SAXS and WAXS data.
